# Antioxidant Responses and Phytochemical Accumulation in *Raphanus* Species Sprouts through Elicitors and Predictive Models under High Temperature Stress

**DOI:** 10.3390/antiox13030333

**Published:** 2024-03-08

**Authors:** María-Trinidad Toro, Roberto Fustos-Toribio, Jaime Ortiz, José Becerra, Nelson Zapata, María Dolores López-Belchí

**Affiliations:** 1 School of Nutrition and Dietetics, Faculty of Medicine and Health Sciences, Universidad Mayor, Temuco 4801043, Chile; maria.toror@umayor.cl; 2 Department of Plant Production, Faculty of Agronomy, Universidad de Concepción, Chillán 3780000, Chile; nzapata@udec.cl; 3 Department of Metallurgical Engineering, Faculty of Engineering, Universidad de Concepción, Concepción 4070386, Chile; robertofustos@udec.cl; 4 Department of Food Science and Chemical Technology, Faculty of Chemical and Pharmaceutical Sciences, University of Chile, Santiago 8330111, Chile; jaortiz@uchile.cl; 5 Natural Products Chemistry Laboratory, Department of Botany, Faculty of Natural and Oceanographic Sciences, University of Concepción, Concepción 4070386, Chile; jbecerra@udec.cl

**Keywords:** antioxidant mechanisms, methyl jasmonate, artificial neural network, abiotic stress

## Abstract

Crop production is being impacted by higher temperatures, which can decrease food yield and pose a threat to human nutrition. In the current study, edible and wild radish sprouts were exposed to elevated growth temperatures along with the exogenous application of various elicitors to activate defense mechanisms. Developmental traits, oxidative damage, glucosinolate and anthocyanin content, and antioxidant capacity were evaluated alongside the development of a predictive model. A combination of four elicitors (citric acid, methyl jasmonate—MeJa, chitosan, and K_2_SO_4_) and high temperatures were applied. The accumulation of bioactives was significantly enhanced through the application of two elicitors, K_2_SO_4_ and methyl jasmonate (MeJa). The combination of high temperature with MeJa prominently activated oxidative mechanisms. Consequently, an artificial neural network was developed to predict the behavior of MeJa and temperature, providing a valuable projection of plant growth responses. This study demonstrates that the use of elicitors and predictive analytics serves as an effective tool to investigate responses and enhance the nutritional value of *Raphanus* species sprouts under future conditions of increased temperature.

## 1. Introduction

The genus *Raphanus*, belonging to the *Brassicaceae* family, comprises two species: *Raphanus sativus* L. (edible radish) and *Raphanus raphanistrum* L. (wild radish) [1]. *R. sativus* is an economically significant vegetable widely cultivated worldwide due to its excellent climatic adaptability and high nutritional value. On the other hand, the wild edible plant *R. raphanistrum* is often cultivated for animal feed or considered a weed in certain areas, but it is also highlighted for its significance in human nutrition. It serves both medicinal purposes and as a food source. In specific cultures, its edible roots and leaves, renowned for their distinctive spicy flavor, have been traditionally consumed [2]. The *Brassicaceae* family stands out as unique because it is a rich source of glucosinolates (GSLs), secondary metabolites containing nitrogen and sulfur compounds. These compounds are almost exclusively present in plants of this family and play a crucial role in plant defense [3,4]. GSLs and their hydrolysis products, isothiocyanates (ITCs), have been reported to play a significant role in cancer chemoprevention in various cellular and animal models. This is likely due to their ability to induce phase 2 detoxification enzymes [5,6]. In plant cells, GSLs are separated from myrosinases, enzymes responsible for their hydrolysis. Myrosinases act when there is a disturbance in the tissue, such as through the chewing of fresh plants by both animals and humans. The enzyme β-D-thioglucosidase, present in the intestinal microbiota, is largely responsible for converting ingested GSLs into its ITCs and related indoles—biologically active molecules with attributed health benefits [7,8]. Furthermore, the *Brassicaceae* family contains a variety of nutrients and phytochemicals, including folic acid, vitamins (C and D), fiber, carotenoids, chlorophyll, and a high concentration of bioactive phenolic compounds, such as anthocyanins. These components have established roles in the prevention of various chronic diseases [9]. In particular, anthocyanins belong to the flavonoid family and are pigments present in different plant organs, such as fruits, flowers, and leaves. Anthocyanins function as antioxidants and confer tolerance to different biotic and abiotic stresses. In *Brassica*, the anthocyanins are mainly acylated cyanidins. Acylation of the anthocyanin molecule improves its stability through intramolecular and/or intermolecular copigmentation and self-association reactions [10], allowing vegetables rich in acylated anthocyanins to serve as a stable source for applications in the food industry.

Under normal conditions, the synthesis of secondary metabolites in plants is limited. However, under stress conditions, they accumulate a large number of these compounds, which can be increased or triggered through elicitors that induce physiological and morphological changes related to defense mechanisms [3,11,12,13]. Various negative abiotic factors in the environment, such as high temperatures, induce stress in plants, and heat waves are expected to be more frequent, last longer, and increase in intensity [14]. This ultimately affects crop yield and the biosynthesis and regulation of secondary metabolites. Some research indicates that secondary compounds increase with controlled high temperatures [11]. Heat stress generally induces the accumulation of reactive oxygen species (ROS) and the activation of detoxification systems [15]. If the temperature is too high, it triggers oxidative bursts that lead to a wide spectrum of responses. Many physiological processes are slowed down or impaired, inducing the accumulation of antioxidants, which protect the cell membrane from breakdown and peroxidation until senescence. Therefore, it is important to achieve a balance between plant growth development and defense mechanisms. Studies have demonstrated that GSL content and other bioactive compounds in *Brassica* species increase at higher temperatures [16,17]. 

The study of sprouts is crucial since it helps us to dilucidate the complex processes of germination and early plant growth, providing critical insights into the beginning of plant development, such as essential biological mechanisms. In addition, it shows the key to enhancing agricultural practices, fostering sustainable food production, and addressing global challenges, such as food security and environmental sustainability. Numerous research investigations have demonstrated that sprouts cultivated from seeds in the germination phase possess significant nutritional, biological, and medicinal benefits, indicating an increase in the antioxidant, antidiabetic, anti-inflammatory, hypolipidemic, and anticarcinogenic properties of plant extracts [18]. Sprouts and microgreens exhibit elevated levels of bioactive compounds compared with mature plants, attributed to the dispersion and expansion of phytochemicals caused by the tissue growth of plants in their later developmental stages [19,20].

Hence, the purpose of this study was to evaluate the influence of different elicitors (chitosan, citric acid, methyl jasmonate, and potassium sulfate) on edible radish and wild radish sprouts at a higher growth temperature (up to 10 °C above). This evaluation includes morphological and physiological aspects and bioactive compounds of the food sprouts, such as the content of total and individual GSLs and individual anthocyanins, total phenolic compounds, and antioxidant capacity. This is followed by an evaluation of the oxidative damage induced by the MDA assay, as it is one of the most used indicators of lipid peroxidation. Considering the complexity of the interaction, a principal component analysis (PCA) was used to assess the conditions (type of elicitor) capable of providing the most significant overall effect on biomass and the phytochemical composition of edible radish and wild radish sprouts. Additionally, to optimize the elicitation process, a multivariate analysis tool (artificial neural networks—ANNs) was applied to evaluate the response of the sprouts to high temperatures in combination with the hormone methyl jasmonate in terms of antioxidant capacity (DPPH and ORAC), total phenolic content (TPC), and oxidative stress (MDA). This research aims to innovate in the agri-food industry and explore the behavior of *Raphanus* species exposed to high-temperature conditions and foliar application treatments in relation to the production and accumulation of phytochemicals, addressing the issue of food security.

## 2. Materials and Methods

### 2.1. Plant Material and Sprout Treatments

Edible radish (ER) seeds obtained from Semillería San Alfonso SL (Santiago, Chile) and wild radish (WR) seeds from Lautaro city, Araucanía Region, Chile (Latitude: −38.5167, Longitude: −72.45, 38°31′0″ South, 72°27′0″ West) were utilized in this study. The seeds underwent soaking in sodium hypochlorite and distilled water, followed by weighing and spreading in trays with coconut fiber as a substrate. Four replicates per treatment were positioned in a controlled environment chamber with a 16 h light to 8 h dark cycle and air temperatures of 20/15 °C (optimum) or 30/24 °C (higher). Relative humidity (RH) was maintained at 60% during the day and 80% at night, with photosynthetically active radiation (PAR) set at 400 μmol m^−2^ s^−1^. Chitosan demonstrates elicitor activity, bolstering plant defense by stimulating phytoalexin production, suggesting its potential application to enhance the biosynthesis of bioactive metabolites in plants. The solution was prepared in acetic acid at a concentration of 1% (*w*/*v*) and subsequently diluted to attain final concentrations of 150 mg/L [21]. Citric acid and potassium sulfate (K_2_SO_4_) were dissolved in milli-Q water at concentrations of 5 mM [22] and 3 mM [23], respectively. The utilization of citric acid as an elicitor remains a relatively unexplored research area with limited available information. K_2_SO_4_ has been shown to stimulate secondary metabolism, resulting in elevated levels of GSLs and phenolic compounds. Methyl jasmonate (MeJA) is a significant cellular regulator involved in plant defense mechanisms in response to stress. MeJA was dissolved in 0.2% ethanol and applied at a concentration of 50 µM [24]. A volume of 20 mL of treatments was applied through exogenous spraying (not as a soaking solution treatment), with two controls prepared at 20 °C (C_1_) and 30 °C (C_2_), each receiving 20 mL of milli-Q water, respectively. During the initial two days, all trays were maintained under controlled dark conditions to promote stem elongation. Subsequently, sprouts were collected seven days after germination (mid-light period) for analysis. All samples were weighed and frozen at −80 °C prior to analysis.

### 2.2. Physical Parameters and Sprouting Potential

To assess the influence of elicitors on the physical properties of ER and WR sprouts at the experiment’s conclusion, measurements of fresh weight (g) and germination rate (%) were taken, the latter following the method described by Ling [25]. Radicle length (cm) was determined by measuring from the tip of the radicle to the base of the hypocotyl using a measuring ruler. Hypocotyl length (cm) of sprouts was determined by measuring the length of the hypocotyl with a Vernier caliper. The latter two measurements were taken daily.

### 2.3. Sprout Stress Response to Elicitors

Freshly germinated sprouts (100 mg) were crushed using a mortar. The homogenized tissue powder was suspended in 1.5 mL of 0.1% TCA and centrifuged at 12,000× *g* for 20 min at 4 °C. A 0.5 mL aliquot of the supernatant was combined with 0.5 mL of a solution containing 20% TCA and 0.5% thiobarbituric acid (TBA). The mixture was heated at 95 °C in a constant-temperature water bath for 30 min and then cooled on ice to ambient temperature. After centrifugation at 12,000× *g* for 15 min, the supernatant was used to measure absorbances at 440 nm, 532 nm, and 600 nm. The MDA concentration was calculated using its extinction coefficient of 155 mM^−1^ [26,27]. The assays were conducted using a 96-well microplate in a Synergy H1 hybrid multi-mode reader (Biotek, Winooski, VT, USA). All reagents were acquired from Merck (Darmstadt, Germany). Lipid peroxidation rate equivalents were expressed as nmol MDA mg^−1^ FW (fresh weight).

### 2.4. Extraction of Bioactive Compounds

Bioactive compounds, specifically GSLs and anthocyanins, were extracted utilizing the techniques described by López through various procedures [28]. GSL extraction involved the use of 1 mL of 70% (*v*/*v*) boiling methanol with freeze-dried samples (50 mg). The samples underwent heating at 70 °C for 30 min in a shaking heating bath, with intermittent shaking every 5 min. The extraction process was stopped by transferring the reaction mixture to an ice-water bath for 5 min. Subsequently, the extracts were centrifuged at 17,500× *g* for 5 min, and the supernatants were filtered through a 0.45 μm PVDF filter. All samples were preserved at −20 °C until further analysis.

For anthocyanins extraction, 0.5 g of each sample was mixed with 5 mL of 25:24:1 (methanol:water:formic acid), stirred for 5 min, and then subjected to ultrasonic treatment for 1 h, followed by overnight refrigeration at 4 °C [28]. The samples were then centrifuged at 10,000 rpm for 10 min and filtered through a 0.22 μm PVDF membrane (Millex V13, Millipore, Bedford, MA, USA) before being transferred to amber vials for chromatographic examination. All solvents utilized in the extraction processes were of analytical grade and procured from Merck (Darmstadt, Germany).

### 2.5. Analysis of Glucosinolates and Anthocyanins

Glucosinolates (GSLs) were identified using standards, and their UV–Vis spectra and retention times were determined by HPLC-DAD, following the methodology established by Baenas [7], where the identification of their fragmentation patterns (M- and MSn) had been previously conducted. Chromatograms were monitored at a wavelength of 227 nm, and intact glucosinolates (GSLs) were quantified with glucoerucin and glucobrassicin (Sigma Aldrich, St. Louis, MO, USA) serving as external standards for aliphatic and indolic GSLs, respectively. The quantification process was carried out in triplicate, and the outcomes were reported as milligrams per 100 g of dry weight (mg 100 g^−1^ DW). In the case of anthocyanin analysis, the identification was conducted using the HPLC-DAD-ESI-MSn system under established conditions for these compounds in cruciferous sprouts [29]. The quantification of the extracted samples was performed using a Hitachi HPLC-DAD system (Hitachi Technologies, MERCK, Darmstadt, Germany) under identical chromatographic conditions, with chromatograms being recorded at 520 nm. Cyanidin 3-O-glucoside was employed as an external standard (Sigma-Aldrich, St. Louis, MO, USA). Similar to the GSL analysis, the anthocyanin analysis was carried out in triplicate, and the results were expressed as milligrams per 100 g of dry weight (mg 100 g^−1^ DW).

### 2.6. Antioxidant Properties

#### 2.6.1. Total Phenolic Contents

The total phenolic content (TPC) of the extracts was analyzed according to the method outlined by Singleton and Rossi [30]. In a microplate reader, Folin–Ciocalteu reagent (0.5 N) was mixed with extract solutions and purified water. After shaking for 30 s, the mixture was incubated for 5 min at 25 °C in the dark. Subsequently, 10% Na_2_CO_3_ was added, shaken for 30 s, and incubated for 1 h at 25 °C in the dark. The absorbance at 750 nm was then measured, and the TPC of the extracts was expressed as mg GAE g^−1^.

#### 2.6.2. Antioxidant Capacity

Both the oxygen radical absorbance capacity (ORACFL) assay [31] and the 2,2-diphenyl-1-picrylhydrazyl (DPPH·) method [32] were employed to measure free radical scavenging activities. In brief, the antioxidant capacity for ORACFL was assessed by measuring the variation in fluorescence after 120 min of reaction with the radical. DPPH was evaluated by measuring the variation in absorbance at 515 nm after 30 min of reaction with the radical. The assays were conducted using a microplate reader as previously described. The results were expressed as μmol Trolox g^−1^, and four replications were carried out.

### 2.7. Statistical Analysis

To analyze the collected results for both species, an analysis of variance (ANOVA) was employed to observe significant differences. Prior to this analysis, the normal distribution conformity of the data at each collection point was examined using the Shapiro–Wilk test. Additionally, Tukey’s post hoc test was performed. Results were considered significant at *p* < 0.05. To elucidate the correlation between variables and discriminate the contents of GSLs, anthocyanins, and other characteristics under different elicitation conditions in the two species, principal component analysis (PCA) was conducted on mean-centered data using R software, with the ggplot2 package utilized for plotting. For a more accurate prediction of the variables’ behavior, such as antioxidant capacity (DPPH and ORAC), total phenolic content (TPC), and the oxidative stress biomarker malondialdehyde (MDA), a three-layer neural network (ANN) was applied. This network comprises an input layer representing the number of input parameters (i.e., temperature, *Raphanus* species, and the MeJa elicitor), an output layer, and a middle or hidden layer. In an ANN model, each neuron aggregates the weighted inputs from different paths and then applies a transfer function to the sum. The resulting value is then directed via output paths to other neurons, forming a series of layers known as multilayer perceptrons [33]. All statistical analyses were performed using R software (v. 4.0.5).

## 3. Results

### 3.1. Sprouts Growth Performance and Malonyl-Dialdehyde Status Affected by Stress 

**Figure 1:**
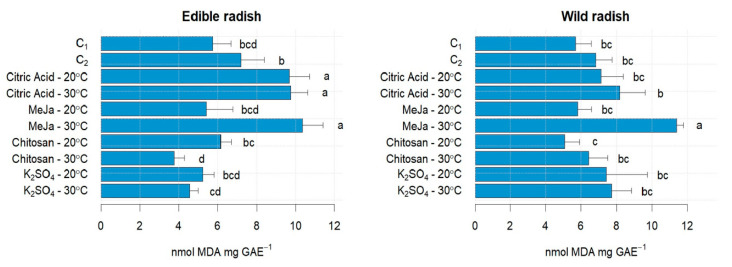
Oxidative stress (MDA content) in sprouts of ER and WR. C_1_: Control at 20 °C; C_2_: Control at 30 °C; Citric Acid—20 °C represents the citric acid elicitor treatment combined with temperature at 20 °C; Citric Acid—30 °C represents the citric acid elicitor treatment combined with temperature at 30 °C; MeJa—20 °C represents the MeJa elicitor treatment combined with temperature at 20 °C; MeJa—30 °C represents the MeJa elicitor treatment combined with temperature at 30 °C; Chitosan—20 °C represents the chitosan elicitor treatment combined with temperature at 20 °C; Chitosan—30 °C represents the chitosan elicitor treatment combined with temperature at 30 °C; K_2_SO_4_—20 °C represents the sulphate potassium elicitor treatment combined with temperature at 20 °C; K_2_SO_4_—30 °C represents the sulphate potassium elicitor treatment combined with temperature at 30 °C. Different letters mean significant differences at *p* < 0.05 in treatments for radish sprouts analyzed separately (edible and wild radish) according to Tukey test.

Morphological parameters were evaluated during 7-day germination, including radicle and hypocotyl growth, fresh weight, and germination index (Supplementary Tables S1 and S2, Figures S1 and S2). All treatments showed radicle emergence and significant elongation over time. After 7 days of sprouting, improving these parameters, an increase of 46% and 6% was observed up to the length of the radicle in ER and WR, respectively (Figure S1A and Figure S1B) and an increase of 67% and 39% in the hypocotyl (ER and WR, respectively) (Figure S1C and Figure S1D) compared with C_2_. Applying high temperature (30 °C) to chitosan resulted in greater hypocotyl and radicle lengths from day 5 of sprouting, with a significant interaction between indicators (*p* < 0.05) in both species. The citric acid elicitor at a high temperature (30 °C) significantly affected plant biomass and germination index, with total biomass production being 3 times greater in the ER species [34] (Figure S2A–D). The ER species obtained high germination rates (greater than 70%) in all treatments. Citric acid had positive effects beyond pH modulation, and higher temperature had positive effects on the morphological characteristics of the sprouts [35]. During elicitation, increased cellular activities lead to the accumulation of reactive oxygen species (ROS) above physiological levels [36]. MDA was used as an indicator of the degree of membrane lipid peroxidation, which is caused by higher levels of reactive oxygen species (ROS) [37,38]. The treatments with MeJa/30 °C and citric acid at 20 °C and 30 °C had a significant effect on MDA compared with C_1_ and C_2_ in ER (*p* < 0.05) (Figure 1), being approximately 40% higher than that of the controls. In the case of WR, only the MeJa/30 °C treatment showed a significant effect, with the MDA content being 67% higher compared with C_2_ (Figure 1). 

### 3.2. Bioactive Compounds in ER and WR Sprouts

#### 3.2.1. Glucosinolates

The 7-day-old radish sprouts showed different GSL profiles (Table 1). GSLs ranged from 51.01 to 81.77 mg 100 g^−1^ DW, and from 44.82 to 103.54 mg 100 g^−1^ DW in ER and WR, respectively.

**Figure 2:**
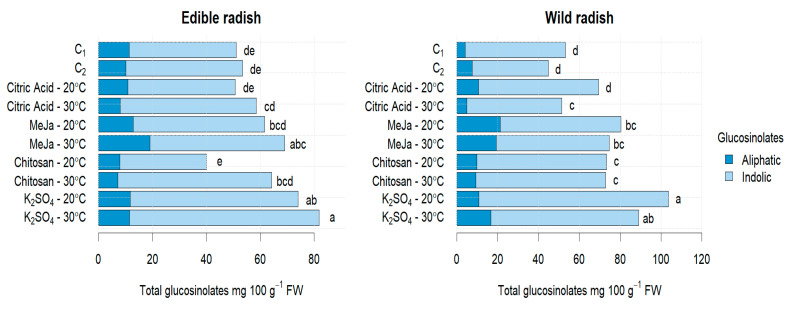
GSLs (aliphatic and indole glucosinolates) in sprouts of ER and WR under the application of different elicitors. C_1_: Control at 20 °C; C_2_: Control at 30 °C; Citric Acid—20 °C represents the citric acid elicitor treatment combined with temperature at 20 °C; Citric Acid—30 °C represents the citric acid elicitor treatment combined with temperature at 30 °C; MeJa—20 °C represents the MeJa elicitor treatment combined with temperature at 20 °C; MeJa—30 °C represents the MeJa elicitor treatment combined with temperature at 30 °C; Chitosan—20 °C represents the chitosan elicitor treatment combined with temperature at 20 °C; Chitosan—30 °C represents the chitosan elicitor treatment combined with temperature at 30 °C; K_2_SO_4_—20 °C represents the sulphate potassium elicitor treatment combined with temperature at 20 °C; K_2_SO_4_—30 °C represents the sulphate potassium elicitor treatment combined with temperature at 30 °C. Different letters mean significant differences at *p* < 0.05 in treatments for radish sprouts analyzed separately (edible and wild radish) according to Tukey test.

All elicitors contributed to the enhancement of total glucosinolates (TGLs), with the elicitors K_2_SO_4_ and MeJa exhibiting a significant effect (*p* < 0.05) at both temperatures (Table 1). Furthermore, the combined application of both resulted in an increased abundance of both indole and aliphatic GSLs. Consistent findings were reported by Hassini in broccoli seedlings [23]. The highest total GSL content was achieved with K_2_SO_4_/30 °C and K_2_SO_4_/20 °C, leading to an increase of approximately 53% and 89% for ER and WR, respectively, compared with C_2_ and C_1_. The predominant GSL identified was glucoraphenin (GRE), constituting 70% and 79% of the total GSLs content in ER and WR, respectively, after 7 days of sprouting. This observation aligns with results reported by other authors [7,28,39]. Concerning GSL composition, distinct responses were noted (Figure 2). Aliphatic GSLs prevailed, accounting for 82% and 84% in ER and WR, respectively, while indole GSLs were present in lower proportions (18% and 16% in ER and WR, respectively).

In both species, K_2_SO_4_, chitosan, and citric acid emerged as the primary elicitors inducing the synthesis of aliphatic GSLs, particularly glucoraphenin (GRE). Conversely, MeJa appeared to be more efficient in promoting the synthesis of indole GSLs, predominantly glucobrassicin (GB). MeJa also demonstrated significant effects on total glucosinolate (TGL) synthesis in both species. In ER, the application of MeJa/30 °C led to a roughly 29% increase compared with C_2_, while in WR, MeJa/20 °C resulted in an approximately 51% increase compared with C_1_. The literature findings support this, showing that MeJa induces GSL accumulation, surpassing 22% content with MeJA treatments in broccoli sprouts [21]. The exogenous application of MeJA (50–250 μM) has been reported to significantly increase TGLs in broccoli and radish sprouts [7]. However, MeJa’s efficiency in indole GSL production was accompanied by a slight decrease in aliphatic GSLs, as reported by Hassini [24] in cabbage sprouts. Chitosan also influenced TGL accumulation in both species, increasing by 20% in ER compared with C_2_ and by 38% in WR compared with C_1_. Chitosan’s impact was more pronounced in the synthesis of aliphatic GSLs. Widely used in agriculture for its ability to induce the biosynthesis of protective biomolecules against pests and pathogens, chitosan also acts as a positive regulator of defensive genes [40]. Notably, Seom Sin [17] reported higher GSL contents in kimchi cabbage plants subjected to extreme high-temperature treatment with the foliar application of chitosan. Citric acid demonstrated a modest increase in TGL accumulation. In ER, it increased by only 9% compared with C_2_, and in WR, it increased by 30% compared with C_1_. These data represent a novel finding, as there are limited reports on the impact of citric acid fertilization on GSL content in vegetable crops to date. 

#### 3.2.2. Anthocyanins

The contents of individual anthocyanins were consistent with data from the literature. The total anthocyanin content (TAC) in sprouts of ER ranged between 6.94 and 0.62 mg 100 g^−1^ of DW and between 5.64 and 1.39 mg 100 g^−1^ DW in sprouts of WR (Table 2). 

Notably, not all elicitors exhibited a positive impact on total anthocyanin content (TAC); however, MeJa addition significantly enhanced TAC abundance. The highest TAC levels were achieved with MeJa/20 °C and MeJa/30 °C treatments for both ER and WR, resulting in a remarkable increase of 60% and 161%, respectively, compared with C_1_ and C_2_. These findings align with Baenas [7], who reported a 23% increase in TAC in radish sprouts with MeJA (25 μM) application. In both species, the prevalent anthocyanin compounds included cyanidin 3-O-(p-coumaroyl) sophoroside-5-O-(malonyl) glucoside, cyanidin 3-O-(feruloyl) sophoroside-5-O-(malonyl) glucoside, and cyanidin 3-O-(sinapoyl) sophoroside-5-O-(malonyl) glucoside, constituting 56% and 73% of TAC in ER and WR under optimum conditions, respectively. These anthocyanins, characterized by three distinct aromatic groups (p-coumaroyl, feruloyl, and sinapoyl) in the diglycosidic substituent at C-3 and an aliphatic group (malonic acid) in the C-5 sugar, mirror similar compositions found in China rose radish sprouts [29], red cabbage [41], and sango radish sprouts [42]. The impact of temperature and its interaction with elicitors significantly influenced TAC accumulation in both species (*p* < 0.05). This susceptibility to temperature variations suggests that the source of stress prompted alterations in anthocyanin synthesis in the sprouts. Notably, ER exhibited lower TAC accumulation at 30 °C, indicating lesser resistance to temperature rise, whereas WR displayed increased TAC synthesis with rising temperature, showcasing greater susceptibility to this environmental change.

### 3.3. Antioxidant Properties of ER and WR Sprouts

**Figure 3:**
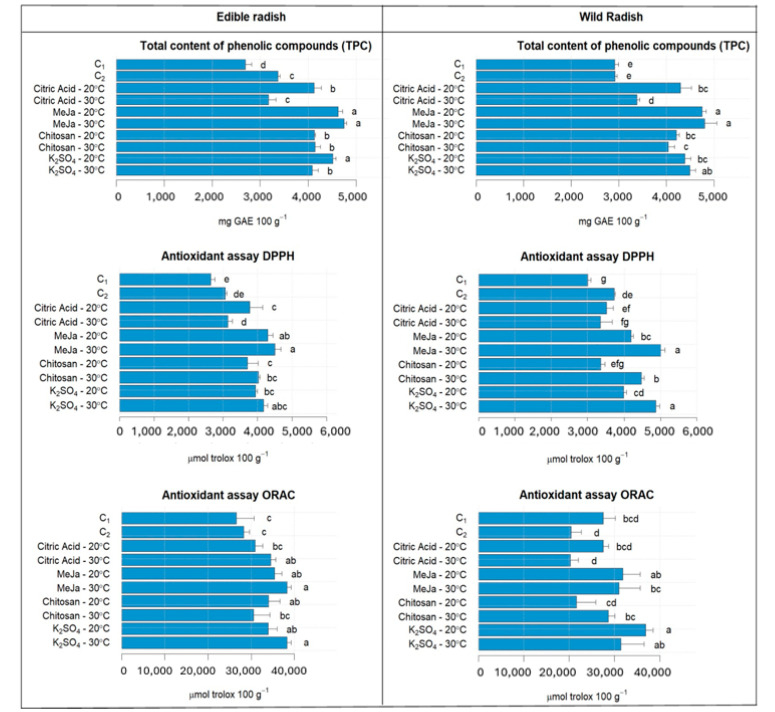
Total phenolic content (TPC) and antioxidant capacity (DPPH and ORAC) assays in ER and WR sprouts. C_1_: Control at 20 °C; C_2_: Control at 30 °C; Citric Acid—20 °C represents the citric acid elicitor treatment combined with temperature at 20 °C; Citric Acid—30 °C represents the citric acid elicitor treatment combined with temperature at 30 °C; MeJa—20 °C represents the MeJa elicitor treatment combined with temperature at 20 °C; MeJa—30 °C represents the MeJa elicitor treatment combined with temperature at 30 °C; Chitosan—20 °C represents the chitosan elicitor treatment combined with temperature at 20 °C; Chitosan—30 °C represents the chitosan elicitor treatment combined with temperature at 30 °C; K_2_SO_4_—20 °C represents the sulphate potassium elicitor treatment combined with temperature at 20 °C; K_2_SO_4_—30 °C represents the sulphate potassium elicitor treatment combined with temperature at 30 °C. Different letters mean significant differences at *p* < 0.05 in treatments for radish sprouts analyzed separately (edible and wild radish) according to Tukey test.

The observed increase in anthocyanin content following MeJa elicitor application in both ER and WR sprouts aligned with the results from the total phenolic content (TPC) and antioxidant capacity assays (Figure 3). Elicitor treatments, across all types, enhanced the antioxidant properties of the sprouts in both species. Specifically, the application of MeJa significantly boosted TPC content. In ER, it rose by 72% (4622.30 mg GAE 100 g^−1^) compared with C_1_ (2693.55 mg GAE 100 g^−1^), and in WR, there was a 63% increase (4747.57 mg GAE 100 g^−1^) compared with C_1_ (2912.66 mg GAE 100 g^−1^). Although K_2_SO_4_ application also increased average TPC content in both species, high temperature exhibited no significant effect on TPC (*p* > 0.05), consistent with findings by De Pascale in *Brassica* rapa species [43]. Conversely, the DPPH antioxidant capacity assay revealed higher values post MeJa/30 °C application in both species. ER showed an increase of 49% (4502.76 µmol Trolox 100 g^−1^) compared with C_2_ (3064.034 µmol Trolox 100 g^−1^), while WR exhibited a 34% increase (5000.57 µmol Trolox 100 g^−1^) compared with C_2_ (3722.63 µmol Trolox 100 g^−1^). As previously reported, MeJa treatment activates the phenylpropanoid pathway, leading to the accumulation of phenolic compounds [12]. Phenolic compounds, known for their antioxidant capabilities, demonstrated higher concentrations in this study, suggesting their role as effective antioxidants. Notably, MeJa and K_2_SO_4_ at 30 °C exhibited no significant difference (*p* > 0.05) in the ORAC assay for ER, increasing by 36% (38,324.47 µmol Trolox 100 g^−1^) compared with C_2_ (28,237.60 µmol Trolox 100 g^−1^). Higher values were observed with the application of K_2_SO_4_/30 °C in WR, increasing by 34% (36,939.03 µmol Trolox 100 g^−1^) compared with C_1_ (27,555.40 µmol Trolox 100 g^−1^).

### 3.4. Interaction of Elicitors with Phytochemical Content: PCA Analysis at 30 °C

**Figure 4:**
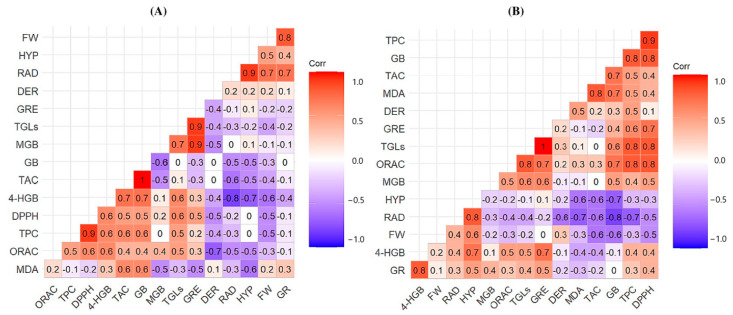
Correlation matrix between different variables at 30 °C, (**A**) correspond to ER and (**B**) correspond to WR. RAD: length of the radicle; HYP: hypocotyl length, FW: weight sprouts; GR: germination rate; MDA: malondialdehyde assay; TGLs: total glucosinolates; TAC: total anthocyanins; GRE: glucoraphenin; 4-HGB: hydroxyglucobrassicin; DER: dehydroerucine; GB: glucobrassicin; MGB: 4-methoxyglucobrassicin; TPC: total phenolic content; DPPH: DPPH assay; ORAC: ORAC assay for ER and WR.

To comprehensively analyze the results globally, a correlation matrix was constructed, as depicted in Figure 4, illustrating the correlations resulting from the application of a combination of elicitors at high temperatures (30 °C). Positive correlations were observed between total phenolic content (TPC) and antioxidant capacity (DPPH and ORAC), attributed to the stress-induced promotion of phenolic compound production, consequently elevating antioxidant capacity. Additionally, both ER and WR species exhibited a positive correlation between total anthocyanin content (TAC) and glucobrassicin (GB). GB also demonstrated a positive correlation with DPPH. Notably, glucoraphenin (GRE) exhibited a high correlation with glucosinolates (GSLs), considering its predominance in both species.

The correlation analysis at 30 °C revealed negative correlations in both species. For instance, parameters such as hypocotyl diameter (HYP), radicle length (RAD), or fresh weight (FW) exhibited a negative correlation with malondialdehyde (MDA) levels, specific hydroxyglucobrassicin (4-HGB), and TAC. Abiotic stress adversely affects plant growth and development by disrupting biochemical and physiological processes, including photosynthesis, respiration, and transpiration. Stressed plants tend to enhance the production of secondary metabolites since growth is often inhibited more than photosynthesis, leading to the predominant allocation of fixed carbon to secondary metabolites [44]. 

Furthermore, a positive correlation between GB and TAC was evident in both species, aligning with the high correlation among GB, TPC, and DPPH. Studies have demonstrated the relationship between GSLs and antioxidant capacity, suggesting that GB could contribute a greater proportion to the overall antioxidant capacity [45]. A noteworthy positive correlation between TAC and MDA was observed in WR at 30 °C. Conversely, observations from the supplementary material (Figure S3) indicated lower correlations in WR at 20 °C, confirming that the combination of high temperatures with elicitor application induced significant oxidative stress in WR, leading to a substantial accumulation of anthocyanins.

**Figure 5:**
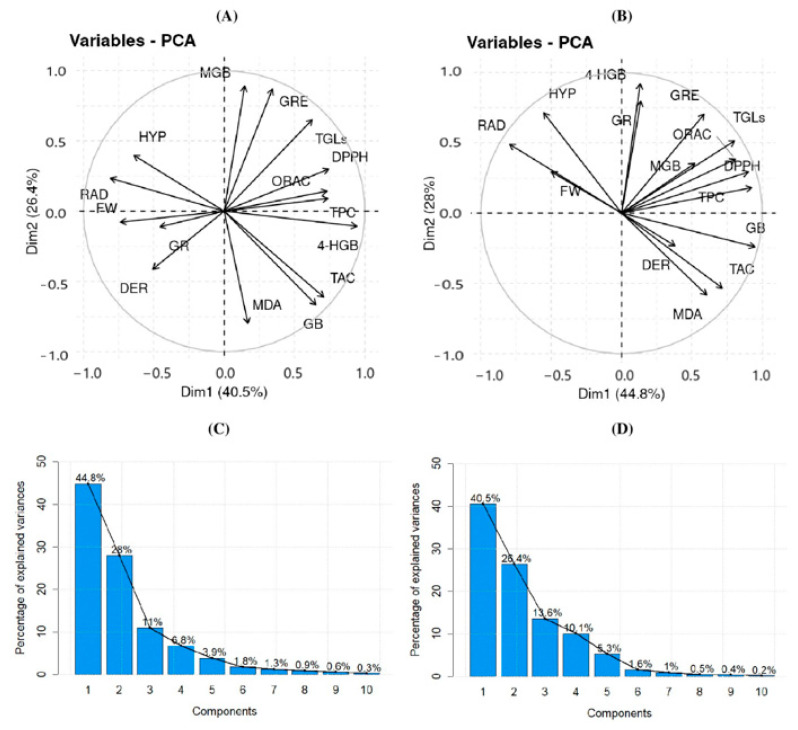
Principal component analysis (PCA) at 30 °C. Letter (**A**) represent the PCA of edible radish, letter (**B**) represent the PCA of wild radish, letter (**C**) represent the percentage of explained of edible radish and letter (**D**) represent the percentage of explained of wild radish. RAD: Length of the radicle; HYP: Hypocotyl length, FW: weight sprouts; GR: germination rate; MDA: Malondialdehyde Assay; TGLs: Total Glucosinolates; TAC: Total anthocyanins; GRE: Glucoraphenin; 4-HGB: Hydroxyglucobrassicin; DER: Dehydroerucine; GB: Glucobrassicin; MGB: 4-methoxyglucobrassicin; TPC: Total phenolic content; DPPH: DPPH assay; ORAC: ORAC assay for ER and WR.

Considering the multitude of variables involved and the observed correlations in the correlation matrix, a comprehensive statistical analysis necessitated a PCA approach to avoid overlooking essential information that could shed light on the underlying experimental processes. To explore the relationship between the original variables and the generated components, a biplot graph was examined at 30 °C (Figure 5), elucidating the first two dimensions (Dim) explaining the variables. Through this rotation, fifteen principal components were derived, mirroring the original variables for both ER and WR. 

For ER at 30 °C (Figure 5A), Dim1 retained 40%, and Dim2 retained 26%. The variables most positively correlated with Dim1 included DPPH, TPC, ORAC, and 4-HGB, while the most negatively correlated variables were FW, RAD, and HYP. Furthermore, PCA at 20 °C (Figure S4A) underscored a negative correlation between GSL 4-HGB and Dim1, in line with the correlation matrix. 

In the case of WR at 30 °C (Figure 5B), Dim1 and Dim2 retained 45% and 28%, respectively. The variables most positively correlated with Dim1 were TPC, DPPH, ORAC, and GB, whereas those negatively correlated were RAD and FW. Dim2 was predominantly correlated with 4-HGB and GR. Conversely, no significant variables negatively related to Dim2 were identified. The PCA conducted at 20 °C for WR (Figure S4) indicated that variables positively correlated with Dim2 were MDA and 4-HGB, whereas TAC showed a negative correlation. In both species, the first three components were concentrated at 80% (Figure 5C) and 84% (Figure 5D), respectively. Thus, focusing on these three variables provides a meaningful reduction, explaining a substantial portion of the observed variation in the fifteen original study variables.

On the other hand, the seedlings of *R. sativus* and *R. raphanistrum*, when reaching 15 days old at 30 °C, revealed notable distinctions in terms of length, dry weight, total glucosinolates, and total anthocyanins compared with their state at 7 days (Tables S3 and S4). However, a consistent trend emerged concerning the various treatments applied. Notably, for both species, seedlings subjected to chitosan treatment displayed increased lengths and dry weights, with the latter effect being particularly pronounced in response to citric acid treatments. Despite the observed outcomes, MeJa treatment exhibited a significantly higher growth ratio, approximately double for *R. sativus*. Nevertheless, there was a concurrent decrease in the content of glucosinolates and anthocyanins over time. Despite this decrease, K_2_SO_4_, chitosan, and MeJa emerged as elicitors associated with elevated content, specifically for glucosinolates, and MeJa for total anthocyanins.

### 3.5. Artificial Neural Networks (ANNs) Modeling to Predict the Impact of High Temperatures and MeJa Exogenous Application

The obtained results reveal that elicitation strategies effectively enhance the bioactive content and biological activity of Raphanus species sprouts under abiotic stress. To evaluate the optimal conditions for achieving the highest accumulation of bioactive compounds, particularly focusing on the MeJa elicitor, mathematical modeling tools have been employed.

**Figure 6:**
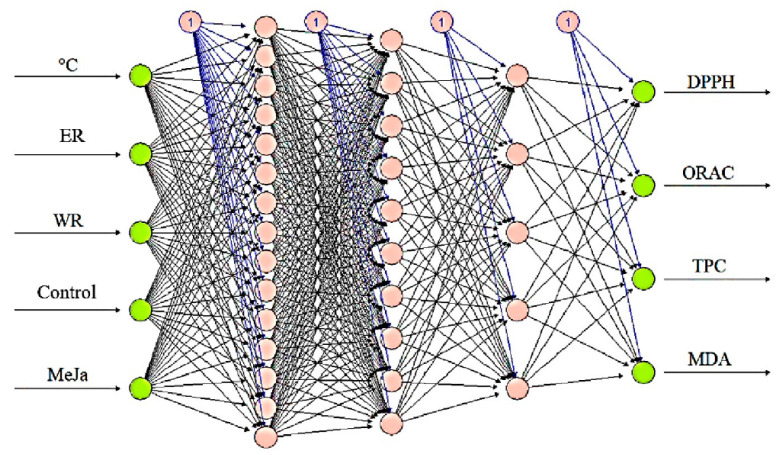
Structure of the ANNs model for the prediction of antioxidant capacity by the DPPH and ORAC method, content of phenolic compounds (TPC), and oxidative stress biomarker malondialdehyde (MDA).

In Figure 6, the architecture of the developed neural network is depicted, comprising an input layer with 4 neurons, three intermediate layers with 15-10-5 neurons, and an output layer with 4 neurons. The ‘back-propagation’ algorithm was utilized to adjust the weights of the intermediate layers, and the network was trained using previously obtained experimental data. The neural network successfully established a correlation of over 90% between fitted and observed values for DPPH, ORAC, TPC, and MDA, demonstrating minimal error values and high prediction accuracy.

For the validation of the ANNs model, the most robust concordance was observed for TPC (R^2^ validation = 0.989), followed by antioxidant activity measured through the DPPH method (R^2^ validation = 0.975) and the MDA test (R^2^ validation = 0.957). The ORAC antioxidant activity assay exhibited the lowest correlation coefficient for validation (R^2^ validation = 0.851) (Figure S5). This underscores the capability of ANNs to accurately predict the optimal biological properties of Raphanus sprouts, as evidenced by the high correlation coefficient between observed and predicted variables. This reliability enhances the trustworthiness of future predictions, contingent upon growth temperature, species, and the applied elicitor.

## 4. Discussion

The elicitors employed in this study (citric acid, MeJa, chitosan, and K_2_SO_4_) are recognized as molecules that activate defense mechanisms in plants, specifically triggering the antioxidant defense system to mitigate excessive ROS production under stress conditions, such as high temperatures. 

Chitosan improved hypocotyl and radicle length. It can enhance plant growth and nutrient uptake since it can serve as an additional source of carbon in plant biosynthetic processes. However, the response varies depending on the species [40,46]. 

The results from this research suggest that elicitor application induced oxidative stress in both ER and WR sprouts, leading to an increase in active oxygen species. To prevent the destruction of cell membrane structure due to heightened ROS levels, elicitation needs to be carefully controlled. The analysis of variance demonstrated a significant (*p* < 0.05) effect of the interaction between elicitors and temperature on MDA content in both species. Sakamoto and Suzuki [47] found that MDA concentration increased with foliar MeJa application on radish sprouts. This situation is explained because MeJa plays a key role in ameliorating oxidative stress, promoting enzyme activities, and increasing the expression levels of defense-related genes in plants [48]. According to Świeca [49], the exposure of plant cells to elevated temperatures (30 °C) induces oxidative stress through the peroxidation of polyunsaturated fatty acids to harmful aldehydes such as MDA.

Concerning the application of K_2_SO_4_, metabolomic and transcriptomic studies have revealed its direct involvement in GSL biosynthesis. Sulfur deficiency in plants results in the reduced expression of major GSL biosynthetic genes, leading to a decrease in GSL production. In *Brassica* species, sulfur nutrition plays a crucial role in determining yield components and seed quality due to their high sulfur requirements throughout the growing season [50]. Studies also indicate that the abundance of sulfur-containing metabolites, including cysteine, glutathione, sulpholipids, and GSLs (aliphatic, indole, and aromatic), decreases due to reduced sulfur availability [51]. Therefore, optimizing sulfur supply could modulate GSL metabolism, although the demand for sulfur in plants varies based on species and developmental stages [52]. For instance, a greater amount of sulfur is required during early vegetative growth [53]. Some studies highlight the positive impact of sulfur-containing amino acid (methionine) fertilization on GSLs content in broccoli sprouts [7], suggesting the potential benefit of utilizing sulfur for GSL synthesis [54].

Jasmonates, known for their role in plant stress management, activate defense mechanisms and various physiological events, including GSL biosynthesis. The exogenous application of MeJA simulates herbivore attack and initiates the jasmonate pathway in plants like broccoli [55]. Studies in Arabidopsis thaliana demonstrate the overexpression of pathway genes, such as IQD1, OBP2, ATR1/MYB34, and HIG1/MYB51, in response to phytohormone application, regulating increased concentrations of major indole GSLs [29,56].

The joint application of K_2_SO_4_ and MeJa significantly improved the accumulation of TGLs and natural antioxidants, including acylated anthocyanins and phenolic compounds, in the sprouts of both species. This improvement was reflected in the values of the peroxidation bioindicator against stress.

Regarding the accumulation of TGLs, ER showed a greater accumulation at high temperatures, while WR was negatively impacted by the temperature increase. In contrast, the content of aliphatic GSLs, such as GRE, known for their potent cytotoxic effects against some cell lines [57], increased due to the temperature rise in both species. A synergistic effect was observed between the elicitor and the temperature increase, with the simultaneous application of both stressors leading to an efficient increase in GRE synthesis. Previous studies have shown that GSL contents, especially aliphatics, increase at higher temperatures (30 °C) [16,58]. Moderately high temperatures (30/24 °C) on kimchi cabbage plants have been reported to markedly improve the content of indole GSLs and aliphatics [17]. Abiotic stresses can alter the GSL profile, with plants under stress enhance the synthesis of secondary messengers that release GSLs from the vacuole into the cytoplasm. This process involves contact with the enzyme myrosinase, leading to hydrolysis and conversion into isothiocyanates, signaling molecules that promote heat tolerance in plants [17,58]. Increased myrosinase activity promotes the content of GSL degradation products, enhancing the plant’s defensive function against stress [59]. However, it has been reported that in radish sprouts treated with ultraviolet light, only about 6% of the GRE compound was converted to sulforaphene, the most important ITC present in radish [60]. This suggests that some GSLs may be metabolized into compounds that do not contribute to ITC synthesis [61]. Elicitation increased the total GSL content, making ER and WR sprouts a prominent source of sulforaphene.

On the other hand, the increase in phenolic compounds in elicitor-treated sprouts can be attributed to de novo synthesis and transformation. One of the pathways for the synthesis of phenolic compounds is through the phenylpropanoid route [12]. Anthocyanin biosynthesis, part of the general phenylpropanoid pathway, involves steps induced by jasmonates. Jasmonates induce the enzyme phenylalanine ammonium lyase (PAL) (EC 4.3.1. 5), a key player in secondary compound formation and sensitive to stress [62]. PAL activity induced by MeJa treatment favors the gene expression of phenolic biosynthesis [21], triggering the accumulation of these secondary metabolites in ER and WR sprouts.

Assessing sprouts instead of mature plants is of significant importance due to several key factors. Sprouts exhibit higher levels of certain health-protecting phytochemicals compared with mature plants, indicating their potential as a rich source of bioactive compounds. Sprouts offer a unique perspective on plant biology, nutrition, and health. Their distinct composition, bioactivity, and nutritional benefits make them a valuable focus of research for enhancing dietary quality, exploring novel applications, and understanding plant responses to environmental stimuli.

The application of a combination of four elicitors (citric acid, methyl jasmonate—MeJa, chitosan, and K_2_SO_4_) along with high temperature has been shown to enhance the accumulation of bioactives in plants. Specifically, the use of two elicitors, K_2_SO_4_ and methyl jasmonate (MeJa), has been found to significantly boost the accumulation of bioactives. This research has demonstrated that mature plants would exhibit similar trends to sprouts when subjected to treatments with signaling molecules, indicating that ontogenetic differences in bioactive compound accumulation may not be as pronounced (Tables S3 and S4). The application of elicitors such as methyl jasmonate has been demonstrated to enhance the accumulation of bioactive compounds in plants. Ref. [63] showed improvements in phenolic contents in grapes through methyl jasmonate application. Additionally, other authors found that the elicitation of different plants with methyl jasmonate and/or ethephon led to changes in secondary metabolites [64,65]. This research indicates that the application of elicitors like methyl jasmonate, in combination with other compounds, can effectively enhance the accumulation of bioactive compounds in plants, regardless of their maturity stage. This highlights the potential of using elicitors to boost the production of valuable phytochemicals in agricultural and pharmaceutical applications. Nevertheless, although sprouts attract attention for their rich concentration of nutrients and bioactive compounds such as antioxidants and glucosinolates, it is crucial to recognize that the nutritional and medicinal benefits of plants are not exclusive to sprouts. Mature plants also contain substantial nutrients and bioactive compounds, providing additional advantages like higher biomass yield and larger-scale production. Conducting research on both sprouts and adult plants can be beneficial in fully optimizing the overall nutritional and medicinal potential of plants.

## 5. Conclusions

The chosen species, edible radish (ER) and wild radish (WR), both belonging to the *Brassicaceae* family, stand out as nearly unique sources rich in GSLs. Notably, this study is the first to investigate the impact of foliar elicitor application in conjunction with elevated temperatures on the secondary metabolite content of the Raphanus species, along with the utilization of predictive models. The application of K_2_SO_4_ and MeJa significantly enhanced the accumulation of TGSLs and natural antioxidants, including acylated anthocyanins and phenolic compounds, in the sprouts of both species. This enhancement was corroborated by values from the peroxidation bioindicator against stress. However, chitosan and citric acid, among the elicitors, demonstrated greater efficiency in improving the physical parameters of the sprouts. The choice between them depends on the specific goals in crop production. Despite observed differences, the impact of elicitors was more pronounced than the effect of a 10-degree temperature increase in terms of sprout growth. The correlation matrices and PCA analysis facilitated the correlation of variables, reducing the original fifteen variables to three, explaining a significant portion of the study’s outcomes. The artificial neural network emerged as a valuable tool for predicting the behavior of DPPH, ORAC, TPC, and MDA variables. Species, MeJa application, and temperature increase influenced antioxidant activity and oxidative damage, with optimal production limited to a temperature range of 20 to 30 °C. Continuous training is essential for the network to enhance understanding and improve predictions at different temperatures. This study underscores the necessity for further research in this domain. Given the interest in bioactive compounds with biological potential, the variation in phytochemical content must be carefully considered. The diverse biological potentials of derivatives of bioactive compounds highlight the importance of identifying, quantifying, and individually determining them in plants and plant tissues. Lastly, this research aims to innovate in the agri-food industry by exploring the behavior of *Raphanus* species exposed to high-temperature conditions and foliar application treatments, contributing to the production and accumulation of phytochemicals essential for food security. Furthermore, this study demonstrates that elicitors, combined with predictive analysis, serve as an effective tool to enhance the nutritional value of Raphanus species sprouts under the anticipated future conditions of increased temperatures.

## Figures and Tables

**Table 1:** Comparison of the content of GSLs of ER and WR after foliar application of the combination of different elicitors and high temperatures.

Edible Radish							
Treatments		Glucosinolates (mg 100 g ^−1^ DW)					
Elicitor	Temperature	GRE	4-HGB	DER	GB	MGB	TGLs
C_1_	20 °C	32.17 ± 7.016 cd	5.20 ± 0.928 bc	7.52 ± 0.627 ab	2.93 ± 0.473 e	3.19 ± 0.545 a	51.01 ± 9.588 de
C_2_	30 °C	34.92 ± 8.018 cd	4.92 ± 0.589 bcd	8.49 ± 0.990 a	4.41 ± 0.285 d	0.78 ± 0.303 c	53.44 ± 9.233 de
Citric acid	20 °C	34.56 ± 3.285 cd	5.17 ± 0.918 bc	5.34 ± 0.147 d	2.85 ± 0.377 e	2.81 ± 0.115 a	50.72 ± 4.842 de
Citric acid	30 °C	43.38 ± 3.421 bc	3.96 ± 0.091 cd	7.19 ± 0.743 abc	2.46 ± 0.324 e	1.56 ± 0.212 bc	58.55 ± 4.791 cd
MeJa	20 °C	41.05 ± 4.536 bc	3.01 ± 0.793 d	7.63 ± 0.499 ab	8.03 ± 0.585 b	1.85 ± 0.312 ab	61.56 ± 6.725 bcd
MeJa	30 °C	43.45 ± 3.595 bc	7.84 ± 0.969 a	6.58 ± 0.418 bcd	10.22 ± 0.400 a	1.01 ± 0.325 c	69.10 ± 5.707 abc
Chitosan	20 °C	26.69 ± 3.540 d	3.44 ± 1.189 cd	5.35 ± 0.501 d	2.09 ± 0.408 e	2.418 ± 0.719 ab	39.98 ± 6.358 e
Chitosan	30 °C	49.92 ± 8.414 b	3.40 ± 0.581 cd	7.15 ± 0.812 abc	2.18 ± 0.190 e	1.57 ± 0.267 bc	64.24 ± 6.765 bcd
K_2_SO_4_	20 °C	54.243 ± 2.804 ab	3.05 ± 0.766 d	8.03 ± 0.611 ab	6.29 ± 0.931 c	2.46 ± 0.300 ab	74.08 ± 5.412 ab
K_2_SO_4_	30 °C	64.43 ± 3.062 a	6.78 ± 1.017 ab	5.83 ± 0.262 cd	2.25 ± 0.312 e	2.47 ± 0.251 bc	81.77 ± 4.904 a
Significance							
Temperature (A)		***	***	NS	NS	***	***
Elicitor (B)		***	**	***	***	***	***
A × B		**	***	***	***	***	*
Wild Radish							
Treatments		Glucosinolates (mg 100 g^−1^ DW)					
Elicitor	Temperature	GRE	4-HGB	DER	GB	MGB	TGLs
C_1_	20 °C	46.56 ± 0.265 def	0.95 ± 0.239 d	2.52 ± 0.252 c	2.50 ± 0.565 e	0.73 ± 0.404 d	53.27 ± 1.705 d
C_2_	30 °C	36.17 ± 6.361 f	0.86 ± 0.093 d	1.13 ± 0.272 d	5.33 ± 0.173 cd	1.33 ± 0.231 cd	44.82 ± 7.113 d
Citric acid	20 °C	57.16 ± 5.697 bcd	3.57 ± 0.722 a	1.53 ± 0.601 d	5.93 ± 0.382 c	1.22 ± 0.355 cd	69.42 ± 7.740 c
Citric acid	30 °C	41.92 ± 3.859 ef	1.07 ± 0.510 d	4.58 ± 0.330 a	2.98 ± 0.746 de	0.92 ± 0.304 cd	51.47 ± 5.732 d
MeJa	20 °C	54.65 ± 2.474 cde	1.94 ± 0.520 cd	4.43 ± 0.129 a	17.64 ± 0.876 a	1.74 ± 0.643 bcd	80.01 ± 4.873 bc
MeJa	30 °C	50.97 ± 7.603 cde	1.45 ± 0.680 cd	4.18 ± 0.229 ab	16.41 ± 0.876 a	1.56 ± 0.613 bcd	74.57 ± 9.002 bc
Chitosan	20 °C	60.88 ± 5.338 bc	1.25 ± 0.360 cd	2.61 ± 0.092 c	5.67 ± 1.073 cd	2.90 ± 0.603 a	73.30 ± 7.453 c
Chitosan	30 °C	60.81 ± 5.547 bc	3.83 ± 0.447 a	2.71 ± 0.566 c	4.08 ± 0.336 cde	1.49 ± 0.502 bcd	72.92 ± 7.372 c
K_2_SO_4_	20 °C	89.22 ± 3.996 a	3.37 ± 0.327 ab	3.45 ± 0.415 bc	4.92 ± 0.927 cde	2.59 ± 0.436 ab	103.54 ± 6.073 a
K_2_SO_4_	30 °C	68.60 ± 6.657 b	2.31 ± 0.273 bc	3.70 ± 0.414 ab	12.37 ± 2.051 b	2.03 ± 0.592 abc	89.01 ± 9.988 ab
Significance							
Temperature (A)		***	*	**	*	**	***
Elicitor (B)		***	***	***	***	***	***
A × B		**	***	***	***	**	*

C_1_: represent control 1 (20 °C without elicitor) and C_2_ represent control 2 (30 °C without elicitor). Mean separation within a column followed by different letters are significantly different according to Tukey test at *p* ≤ 0.05. NS, *, **, and *** indicate non-significant, significant at *p* ≤ 0.05, *p* ≤ 0.01, and *p* ≤ 0.001, respectively. GRE is glucoraphenin; 4-HGB is hydroxyglucobrassycin; DER is dehydroerucin; GB is glucobrassycin; MGB is 4-methoxyglucobrassycin.

**Table 2:** Anthocyanin content (mg 100 g^−1^ DW) in ER and WR sprouts after different treatment with elicitors.

Edible Radish										
Treatments		Anthocyanins (mg 100 g^−1^ DW)								
Elicitor	Temperature	1-Cy	2-Cy	3-Cy	4-Cy	5-Cy	6-Cy	7-Cy	8-Cy	TAC
C_1_	20 °C	1.00 ± 0.045 a	0.51 ± 0.030 ab	0.42 ± 0.039 b	1.80 ± 0.152 b	0.27 ± 0.036 bc	0.11 ± 0.012 bc	0.16 ± 0.011 b	0.05 ± 0.012 ab	4.33 ± 0.302 c
C_2_	30 °C	0.08 ± 0.032 d	0.25 ± 0.132 cd	0.12 ± 0.012 b	0.92 ± 0.144 cd	0.69 ± 0.493 a	0.17 ± 0.056 b	NP	NP	2.22 ± 0.768 de
Citric acid	20 °C	0.36 ± 0.042 bcd	0.36 ± 0.062 bc	0.34 ± 0.020 b	1.13 ± 0.296 bc	0.16 ± 0.073 bc	0.08 ± 0.029 bc	0.14 ± 0.039 b	0.05 ± 0.029 ab	2.61 ± 0.624 d
Citric acid	30 °C	0.12 ± 0.031 d	0.12 ± 0.011 d	0.26 ± 0.096 b	0.21 ± 0.062 de	0.03 ± 0.011 c	0.01 ± 0.004 c	0.03 ± 0.022 d	NP	0.78 ± 0.364 f
MeJa	20 °C	0.71 ± 0.101 ab	0.48 ± 0.086 ab	0.82 ± 0.060 a	3.85 ± 0.489 a	0.27 ± 0.021 bc	0.38 ± 0.057 a	0.33 ± 0.053 a	0.07 ± 0.010 a	6.91 ± 0.500 a
MeJa	30 °C	0.31 ± 0.056 cd	0.57 ± 0.017 a	0.40 ± 0.141 b	3.44 ± 0.521 a	0.40 ± 0.113 ab	0.34 ± 0.108 a	0.02 ± 0.012 d	0.01 ± 0.011 cd	5.49 ± 0.696 b
Chitosan	20 °C	0.57 ± 0.153 bc	0.16 ± 0.043 d	0.24 ± 0.031 b	1.09 ± 0.282 bc	0.11 ± 0.017 bc	0.08 ± 0.013 bc	0.14 ± 0.032 b	0.04 ± 0.006 abc	2.42 ± 0.391 d
Chitosan	30 °C	0.17 ± 0.020 d	0.11 ± 0.010 d	0.10 ± 0.018 b	0.14 ± 0.010 de	0.04 ± 0.013 c	0.03 ± 0.013 c	0.03 ± 0.007 d	NP	0.62 ± 0.067 f
K_2_SO_4_	20 °C	0.13 ± 0.032 d	0.25 ± 0.095 cd	0.24 ± 0.081 b	0.78 ± 0.291 cde	0.09 ± 0.037 bc	0.05 ± 0.024 c	0.11 ± 0.029 bc	0.02 ± 0.015 bcd	1.67 ± 0.393 def
K_2_SO_4_	30 °C	0.04 ± 0.015 d	0.13 ± 0.022 d	0.17 ± 0.033 b	0.51 ± 0.091 cde	0.15 ± 0.022 bc	0.10 ± 0.009 bc	0.05 ± 0.010 cd	NP	1.13 ± 0.098 ef
Significance										
Temperature (A)		***	***	**	***	NS	NS	***	***	***
Elicitor (B)		***	***	***	***	***	***	***	**	***
A × B		**	***	NS	NS	*	*	***	NS	*
Treatments		Anthocyanins (mg 100 g^−1^ DW)								
Elicitor	Temperature	1-Cy	2-Cy	3-Cy	4-Cy	5-Cy	6-Cy	7-Cy	8-Cy	TAC
C_1_	20 °C	0.04 ± 0.017 d	0.08 ± 0.025 bc	0.39 ± 0.077 b	1.47 ± 0.342 bc	0.02 ± 0.021 d	0.09 ± 0.011 cd	0.01 ± 0.006 bc	0.06 ± 0.021 ab	2.16 ± 0.426 bcd
C_2_	30 °C	0.28 ± 0.043 ab	0.12 ± 0.033 ab	0.57 ± 0.106 ab	1.86 ± 0.571 b	0.09 ± 0.01 bcd	0.07 ± 0.004 cd	0.04 ± 0.015 ab	0.03 ± 0.004 abc	3.06 ± 0.676 b
Citric acid	20 °C	0.14 ± 0.011 cd	0.04 ± 0.009 c	0.30 ± 0.055 b	1.22 ± 0.276 bcd	0.05 ± 0.014 cd	0.11 ± 0.018 bcd	0.02 ± 0.006 bc	0.01 ± 0.001 cd	1.87 ± 0.367 cd
Citric acid	30 °C	0.35 ± 0.063 a	0.17 ± 0.011 a	0.70 ± 0.145 a	0.86 ± 0.155 cd	0.15 ± 0.019 b	0.18 ± 0.067 ab	0.04 ± 0.013 ab	0.03 ± 0.027 abc	2.50 ± 0.303 bc
MeJa	20 °C	0.14 ± 0.029 cd	0.08 ± 0.025 bc	0.70 ± 0.168 a	3.61 ± 0.360 a	0.12 ± 0.028 bc	0.20 ± 0.056 a	0.07 ± 0.022 a	0.04 ± 0.006 ab	4.96 ± 0.500 a
MeJa	30 °C	0.11 ± 0.015 cd	0.08 ± 0.013 bc	0.75 ± 0.138 a	4.14 ± 0.571 a	0.23 ± 0.086 a	0.22 ± 0.052 a	0.05 ± 0.02 ab	0.06 ± 0.015 a	5.64 ± 0.649 a
Chitosan	20 °C	0.33 ± 0.039 a	0.12 ± 0.011 ab	0.42 ± 0.054 b	0.44 ± 0.093 d	0.05 ± 0.006 cd	0.02 ± 0.006 d	NP	NP	1.39 ± 0.060 d
Chitosan	30 °C	0.27 ± 0.079 ab	0.13 ± 0.047 ab	0.71 ± 0.120 a	0.74 ± 0.159 cd	0.07 ± 0.008 cd	0.14 ± 0.005 abc	0.02 ± 0.005 bc	0.03 ± 0.01 bcd	2.11 ± 0.274 bcd
K_2_SO_4_	20 °C	0.19 ± 0.029 bc	0.08 ± 0.020 bc	0.42 ± 0.047 b	1.45 ± 0.197 bc	0.08 ± 0.017 bcd	0.04 ± 0.005 d	0.03 ± 0.017 abc	NP	2.29 ± 0.231 bcd
K_2_SO_4_	30 °C	0.05 ± 0.021 d	0.08 ± 0.011 bc	0.36 ± 0.058 b	1.38 ± 0.114 bc	0.08 ± 0.013 bcd	0.19 ± 0.046 ab	0.06 ± 0.025 a	NP	2.22 ± 0.079 bcd
Significance										
Temperature (A)		***	***	***	NS	NS	NS	*	NS	*
Elicitor (B)		***	**	***	***	***	***	***	***	***
A × B		***	***	**	*	***	***	**	**	**

Mean separation within a column followed by different letters are significantly different according to Tukey test at *p* ≤ 0.05. NS, *, ** and *** indicate non-significance. Significant at *p* ≤ 0.05., *p* ≤ 0.01, and *p* ≤ 0.001, respectively. NP means not present. 1-Cy is Cy-3-O-(CA)soph-5-O-(MA)glu; 2-Cy is Cy-3-O-(CA-FE)soph-5-O-(MA)glu; 3-Cy is Cy-3-O-(pCoA)soph-5-O-(MA)glu + Cy-3-O-(FE-SI)diglu-5-O-glu; 4-Cy is Cy-3-O-(pCoA)soph-5-O-(MA)glu + Cy-3-O-(FE)soph-5-O-(MA)glu + Cy-3-O-(SI)soph-5-O-(MA)glu; 5-Cy is Cy-3-O-(diSI)soph-5-O-(MA)glu + Cy-3-O-(CA-FE)soph-5-O-(MA)glu; 6-Cy is Cy-3-O-(FE-SI)soph-5-O-(MA)glu + Pg-3-O-(FE)soph-5-O-(MA)glu; 7-Cy is Cy-3-O-(CA-SI)soph-5-O-(MA)glu + Cy-3-O-(CA-FE)soph-5-O-(MA)glu; 8-Cy is Cy-3-O-(diFE)soph-5-O-(MA)glu. Cy = cyanidin; glu = glucoside; SI = sinapoyl; CA = caffeoyl; soph = sophoroside; *p*CoA = *p*-coumaroyl; FE = feruloyl; MA = malonyl.

## Data Availability

The data sets generated during the current study are included in this published article and the Supporting information or they are available from the corresponding author on reasonable request.

## Supplementary Materials

The following supporting information can be downloaded at: https://www.mdpi.com/article/10.3390/antiox13030333/s1, Table S1: Root Development in Edible Radish and Wild Radish Sprouts Over a 7-Day Sprouting Period; Table S2: Desarrollo del hipocótilo en brotes de Rábano comestible y Rábano silvestre durante 7 días de brotación; Table S3. Report on Growth and Bioactive Compound Production in 15-Day-Old Raphanus sativus Seedlings at 30 °C; Table S4. Report on Growth and Bioactive Compound Production in 15-Day-Old Raphanus raphanistrum Seedlings at 30 °C; Figure S1: Effect of elicitors on radicle growth and hypocotyl growth in 7-day germinated sprouts; Figure S2: Effect of elicitors on fresh weight and germination rate in 7-day germinated sprouts; Figure S3: Correlation matrix between different variables at 20 °C, (A) correspond to ER and (B) correspond to WR; Figure S4: Principal component analysis (PCA) at 20 °C; Figure S5. Variables predicted by ANNs v/s observed variables for DPPH, ORAC, TPC and MDA.
