# Supplementary material for: Antioxidant Responses and Phytochemical Accumulation in Raphanus Species Sprouts through Elicitors and Predictive Models under High Temperature Stress
Source: Antioxidants (Basel). 2024 Mar 8;13(3):333. doi: 10.3390/antiox13030333 (PMC10967877; doi:10.3390/antiox13030333)
Supplement: Supplementary file 1 [file antioxidants-13-00333-s001.zip › antioxidants-2889513-supplementary.pdf]

Antioxidant Responses and Phytochemical Accumulation in Raphanus Species Sprouts through Elicitors and Predictive Models under High Temperature Stress

María-Trinidad Toro [1], Roberto Fustos [2], Jaime Ortiz [3], José Becerra [4], Nelson Zapata [5], María-Dolores López [5]*

[1] School of Nutrition and Dietetics, Faculty of Medicine and Health Sciences, Universidad Mayor, Temuco, Chile; maria.toror@umayor.cl
[2] Department of Metallurgical Engineering, Faculty of Engineering, Universidad de Concepción, Concepción, Chile; robertofustos@udec.cl.
[3] Department of Food Science and Chemical Technology, Faculty of Chemical and Pharmaceutical Sciences, University of Chile, Santiago, Chile; jaortiz@uchile.cl.
[4] Department of Botany, Faculty of Natural and Oceanographic Sciences, University of Concepción, Natural Products Chemistry Laboratory, Concepción, Chile; jbecerra@udec.cl
[5] Department of Plant Production, Faculty of Agronomy, Universidad de Concepción, Chillán, Chile, nzpata@udec.cl; mlopezb@udec.cl.
* Correspondence: mlopezb@udec.cl; Tel.: +56-412661456.

Supplementary Materials

Table S1. Root Development in Edible Radish and Wild Radish Sprouts Over a 7-Day Sprouting Period.

Edible radish					
20°C			28°C		
Elicitor	Day	Radicle length (cm)	Elicitor	Day	Radicle length (cm)
Control	1	0.883	Control	1	0.650
Control	1	0.655	Control	1	0.578
Control	1	0.786	Control	1	0.544
Citric Acid	1	0.567	Citric Acid	1	0.58
Citric Acid	1	0.542	Citric Acid	1	0.796
Citric Acid	1	0.489	Citric Acid	1	0.871
MeJa	1	0.387	MeJa	1	0.642
MeJa	1	0.607	MeJa	1	0.523
MeJa	1	0.599	MeJa	1	0.589
Chitosan	1	0.482	Chitosan	1	0.482
Chitosan	1	0.392	Chitosan	1	0.495
Chitosan	1	0.554	Chitosan	1	0.590
K_2SO_4	1	0.733	K_2SO_4	1	0.612
K_2SO_4	1	0.835	K_2SO_4	1	0.633

K_2SO_4	1	0.872	K_2SO_4	1	0.714
Control	2	1.235	Control	2	2.245
Control	2	1.123	Control	2	2.550
Control	2	1.282	Control	2	2.322
Citric Acid	2	1.243	Citric Acid	2	2.044
Citric Acid	2	1.145	Citric Acid	2	1.581
Citric Acid	2	1.163	Citric Acid	2	2.179
MeJa	2	0.968	MeJa	2	2.164
MeJa	2	1.396	MeJa	2	1.258
MeJa	2	1.215	MeJa	2	1.160
Chitosan	2	0.603	Chitosan	2	2.703
Chitosan	2	0.776	Chitosan	2	2.785
Chitosan	2	0.772	Chitosan	2	2.655
K_2SO_4	2	1.584	K_2SO_4	2	2.326
K_2SO_4	2	1.509	K_2SO_4	2	1.945
K_2SO_4	2	2.033	K_2SO_4	2	2.012
Control	3	1.650	Control	3	3.533
Control	3	1.583	Control	3	3.680
Control	3	1.575	Control	3	3.744
Citric Acid	3	2.255	Citric Acid	3	2.842
Citric Acid	3	2.773	Citric Acid	3	2.800
Citric Acid	3	2.687	Citric Acid	3	3.077
MeJa	3	1.032	MeJa	3	2.378
MeJa	3	1.181	MeJa	3	2.271
MeJa	3	1.103	MeJa	3	2.377
Chitosan	3	0.931	Chitosan	3	3.531
Chitosan	3	1.080	Chitosan	3	3.850
Chitosan	3	1.136	Chitosan	3	3.821
K_2SO_4	3	1.881	K_2SO_4	3	2.855
K_2SO_4	3	1.954	K_2SO_4	3	2.745
K_2SO_4	3	2.062	K_2SO_4	3	2.698
Control	4	2.101	Control	4	4.144
Control	4	2.029	Control	4	4.212
Control	4	2.205	Control	4	4.254
Citric Acid	4	2.987	Citric Acid	4	4.874
Citric Acid	4	3.032	Citric Acid	4	4.590
Citric Acid	4	3.033	Citric Acid	4	4.798
MeJa	4	2.658	MeJa	4	3.321
MeJa	4	2.443	MeJa	4	3.476
MeJa	4	2.581	MeJa	4	3.276
Chitosan	4	1.349	Chitosan	4	4.349

Chitosan	4	1.657	Chitosan	4	4.235
Chitosan	4	1.232	Chitosan	4	4.182
K_2SO_4	4	2.864	K_2SO_4	4	3.021
K_2SO_4	4	2.965	K_2SO_4	4	2.987
K_2SO_4	4	2.732	K_2SO_4	4	3.210
Control	5	5.657	Control	5	4.550
Control	5	5.788	Control	5	4.412
Control	5	5.667	Control	5	4.355
Citric Acid	5	3.565	Citric Acid	5	5.237
Citric Acid	5	3.451	Citric Acid	5	5.197
Citric Acid	5	3.478	Citric Acid	5	5.403
MeJa	5	3.265	MeJa	5	3.876
MeJa	5	3.850	MeJa	5	4.061
MeJa	5	3.002	MeJa	5	5.609
Chitosan	5	2.737	Chitosan	5	5.737
Chitosan	5	2.570	Chitosan	5	5.490
Chitosan	5	2.593	Chitosan	5	5.609
K_2SO_4	5	3.324	K_2SO_4	5	3.230
K_2SO_4	5	3.012	K_2SO_4	5	3.451
K_2SO_4	5	3.265	K_2SO_4	5	3.822
Control	6	5.855	Control	6	4.744
Control	6	5.798	Control	6	4.656
Control	6	5.874	Control	6	4.612
Citric Acid	6	3.955	Citric Acid	6	5.237
Citric Acid	6	3.987	Citric Acid	6	5.391
Citric Acid	6	4.004	Citric Acid	6	5.253
MeJa	6	3.754	MeJa	6	4.365
MeJa	6	3.633	MeJa	6	4.474
MeJa	6	3.486	MeJa	6	4.371
Chitosan	6	3.051	Chitosan	6	6.351
Chitosan	6	3.455	Chitosan	6	6.699
Chitosan	6	3.045	Chitosan	6	6.598
K_2SO_4	6	3.874	K_2SO_4	6	3.633
K_2SO_4	6	3.532	K_2SO_4	6	3.766
K_2SO_4	6	3.724	K_2SO_4	6	3.644
Control	7	6.022	Control	7	4.832
Control	7	6.012	Control	7	4.988
Control	7	6.212	Control	7	4.798
Citric Acid	7	5.251	Citric Acid	7	6.265
Citric Acid	7	5.487	Citric Acid	7	6.312
Citric Acid	7	5.368	Citric Acid	7	6.213

MeJa	7	4.076	MeJa	7	4.620
MeJa	7	3.965	MeJa	7	4.590
MeJa	7	4.130	MeJa	7	4.723
Chitosan	7	4.745	Chitosan	7	7.100
Chitosan	7	4.854	Chitosan	7	7.021
Chitosan	7	4.733	Chitosan	7	7.189
K_2SO_4	7	4.462	K_2SO_4	7	4.586
K_2SO_4	7	4.168	K_2SO_4	7	4.590
K_2SO_4	7	4.356	K_2SO_4	7	4.638

Wild radish

20°C			28°C		
Elicitor	Day	Radicle length (cm)	Elicitor	Day	Radicle length (cm)
Control	1	0.682	Control	1	1.255
Control	1	0.533	Control	1	1.124
Control	1	0.781	Control	1	1.036
Citric Acid	1	0.570	Citric Acid	1	1.254
Citric Acid	1	0.426	Citric Acid	1	1.152
Citric Acid	1	0.391	Citric Acid	1	1.332
MeJa	1	0.532	MeJa	1	0.643
MeJa	1	0.607	MeJa	1	0.446
MeJa	1	0.589	MeJa	1	0.564
Chitosan	1	0.376	Chitosan	1	0.556
Chitosan	1	0.489	Chitosan	1	0.577
Chitosan	1	0.408	Chitosan	1	0.699
K_2SO_4	1	0.432	K_2SO_4	1	0.644
K_2SO_4	1	0.656	K_2SO_4	1	0.555
K_2SO_4	1	0.533	K_2SO_4	1	0.421
Control	2	0.529	Control	2	2.765
Control	2	1.274	Control	2	2.845
Control	2	1.002	Control	2	2.754
Citric Acid	2	0.898	Citric Acid	2	2.623
Citric Acid	2	0.973	Citric Acid	2	2.565
Citric Acid	2	0.002	Citric Acid	2	2.632
MeJa	2	1.484	MeJa	2	1.164
MeJa	2	1.232	MeJa	2	1.258
MeJa	2	1.550	MeJa	2	1.160
Chitosan	2	0.854	Chitosan	2	1.033
Chitosan	2	0.837	Chitosan	2	1.237
Chitosan	2	0.672	Chitosan	2	1.261

K_2SO_4	2	0.883	K_2SO_4	2	1.895
K_2SO_4	2	1.244	K_2SO_4	2	1.488
K_2SO_4	2	1.105	K_2SO_4	2	1.633
Control	3	1.780	Control	3	3.012
Control	3	1.850	Control	3	3.324
Control	3	1.736	Control	3	3.114
Citric Acid	3	1.333	Citric Acid	3	2.842
Citric Acid	3	1.243	Citric Acid	3	2.763
Citric Acid	3	2.048	Citric Acid	3	2.852
MeJa	3	1.667	MeJa	3	2.878
MeJa	3	1.720	MeJa	3	2.682
MeJa	3	1.721	MeJa	3	2.512
Chitosan	3	1.032	Chitosan	3	2.791
Chitosan	3	1.281	Chitosan	3	2.882
Chitosan	3	1.273	Chitosan	3	2.755
K_2SO_4	3	1.783	K_2SO_4	3	2.612
K_2SO_4	3	1.555	K_2SO_4	3	2.845
K_2SO_4	3	1.305	K_2SO_4	3	2.780
Control	4	2.773	Control	4	4.966
Control	4	2.550	Control	4	5.021
Control	4	2.636	Control	4	5.111
Citric Acid	4	2.435	Citric Acid	4	4.874
Citric Acid	4	2.051	Citric Acid	4	4.712
Citric Acid	4	2.683	Citric Acid	4	4.764
MeJa	4	1.918	MeJa	4	3.320
MeJa	4	2.319	MeJa	4	3.112
MeJa	4	2.088	MeJa	4	3.212
Chitosan	4	1.667	Chitosan	4	4.225
Chitosan	4	1.863	Chitosan	4	4.256
Chitosan	4	1.960	Chitosan	4	4.087
K_2SO_4	4	2.164	K_2SO_4	4	3.114
K_2SO_4	4	1.883	K_2SO_4	4	3.247
K_2SO_4	4	2.453	K_2SO_4	4	3.304
Control	5	4.970	Control	5	6.020
Control	5	4.863	Control	5	5.988
Control	5	4.655	Control	5	6.140
Citric Acid	5	3.677	Citric Acid	5	5.237
Citric Acid	5	4.054	Citric Acid	5	5.343
Citric Acid	5	3.954	Citric Acid	5	5.454
MeJa	5	2.204	MeJa	5	3.822
MeJa	5	2.372	MeJa	5	3.756

MeJa	5	2.502	MeJa	5	3.732
Chitosan	5	2.662	Chitosan	5	4.500
Chitosan	5	2.722	Chitosan	5	4.471
Chitosan	5	2.836	Chitosan	5	4.572
K2SO4	5	2.643	K_2SO_4	5	3.547
K2SO4	5	2.722	K_2SO_4	5	3.805
K2SO4	5	2.665	K_2SO_4	5	3.845
Control	6	5.044	Control	6	6.647
Control	6	5.112	Control	6	6.487
Control	6	4.988	Control	6	6.544
Citric Acid	6	5.872	Citric Acid	6	5.621
Citric Acid	6	5.502	Citric Acid	6	5.532
Citric Acid	6	5.633	Citric Acid	6	5.843
MeJa	6	2.598	MeJa	6	4.212
MeJa	6	2.632	MeJa	6	4.418
MeJa	6	2.754	MeJa	6	4.271
Chitosan	6	3.154	Chitosan	6	5.781
Chitosan	6	3.002	Chitosan	6	5.612
Chitosan	6	3.040	Chitosan	6	5.589
K_2SO_4	6	3.354	K_2SO_4	6	3.877
K_2SO_4	6	3.754	K_2SO_4	6	4.405
K_2SO_4	6	3.565	K_2SO_4	6	4.134
Control	7	5.422	Control	7	7.210
Control	7	5.390	Control	7	7.221
Control	7	5.530	Control	7	7.321
Citric Acid	7	6.207	Citric Acid	7	6.471
Citric Acid	7	6.306	Citric Acid	7	6.512
Citric Acid	7	6.336	Citric Acid	7	6.321
MeJa	7	3.473	MeJa	7	4.787
MeJa	7	3.235	MeJa	7	4.634
MeJa	7	3.029	MeJa	7	4.834
Chitosan	7	4.814	Chitosan	7	7.665
Chitosan	7	4.960	Chitosan	7	7.726
Chitosan	7	5.051	Chitosan	7	7.764
K_2SO_4	7	4.332	K_2SO_4	7	5.760
K_2SO_4	7	4.464	K_2SO_4	7	5.531
K_2SO_4	7	4.212	K_2SO_4	7	4.620

Table S2. Desarrollo del hipocótilo en brotes de Rábano comestible y Rábano silvestre durante 7 días de brotación.

Edible radish					
20°C			28°C		
Elicitor	Day	Hypocotyl length (cm)	Elicitor	Day	Hypocotyl length (cm)
Control	1	0	Control	1	0
Control	1	0	Control	1	0
Control	1	0	Control	1	0
Citric Acid	1	0	Citric Acid	1	0
Citric Acid	1	0	Citric Acid	1	0
Citric Acid	1	0	Citric Acid	1	0
MeJa	1	0	MeJa	1	0
MeJa	1	0	MeJa	1	0
MeJa	1	0	MeJa	1	0
Chitosan	1	0	Chitosan	1	0
Chitosan	1	0	Chitosan	1	0
Chitosan	1	0	Chitosan	1	0
K_2SO_4	1	0	K_2SO_4	1	0
K_2SO_4	1	0	K_2SO_4	1	0
K_2SO_4	1	0	K_2SO_4	1	0
Control	2	0.602	Control	2	0.850
Control	2	0.707	Control	2	0.865
Control	2	0.556	Control	2	0.843
Citric Acid	2	0.563	Citric Acid	2	0.927
Citric Acid	2	0.482	Citric Acid	2	0.692
Citric Acid	2	0.550	Citric Acid	2	0.817
MeJa	2	0.519	MeJa	2	0.634
MeJa	2	0.627	MeJa	2	0.613
MeJa	2	0.733	MeJa	2	0.648
Chitosan	2	0.713	Chitosan	2	0.550
Chitosan	2	0.742	Chitosan	2	0.558
Chitosan	2	0.683	Chitosan	2	0.633
K_2SO_4	2	0.398	K_2SO_4	2	0.612
K_2SO_4	2	0.724	K_2SO_4	2	0.787
K_2SO_4	2	0.633	K_2SO_4	2	0.888
Control	3	0.981	Control	3	1.020
Control	3	1.003	Control	3	1.109
Control	3	0.906	Control	3	1.043
Citric Acid	3	1.132	Citric Acid	3	1.194
Citric Acid	3	1.273	Citric Acid	3	1.292

Citric Acid	3	1.383	Citric Acid	3	1.371
MeJa	3	0.833	MeJa	3	1.182
MeJa	3	0.779	MeJa	3	1.061
MeJa	3	0.681	MeJa	3	0.921
Chitosan	3	1.233	Chitosan	3	1.891
Chitosan	3	1.102	Chitosan	3	1.783
Chitosan	3	1.038	Chitosan	3	1.955
K_2SO_4	3	1.005	K_2SO_4	3	1.176
K_2SO_4	3	1.002	K_2SO_4	3	1.065
K_2SO_4	3	0.881	K_2SO_4	3	1.186
Control	4	1.473	Control	4	1.303
Control	4	1.632	Control	4	1.275
Control	4	1.344	Control	4	1.321
Citric Acid	4	1.614	Citric Acid	4	1.944
Citric Acid	4	1.806	Citric Acid	4	1.954
Citric Acid	4	1.432	Citric Acid	4	2.191
MeJa	4	1.400	MeJa	4	1.375
MeJa	4	1.139	MeJa	4	1.252
MeJa	4	1.437	MeJa	4	1.309
Chitosan	4	1.716	Chitosan	4	2.572
Chitosan	4	1.694	Chitosan	4	1.954
Chitosan	4	1.718	Chitosan	4	2.201
K_2SO_4	4	1.636	K_2SO_4	4	2.082
K_2SO_4	4	1.226	K_2SO_4	4	1.912
K_2SO_4	4	1.476	K_2SO_4	4	1.823
Control	5	1.746	Control	5	1.531
Control	5	1.806	Control	5	1.465
Control	5	1.788	Control	5	1.554
Citric Acid	5	1.790	Citric Acid	5	2.459
Citric Acid	5	2.012	Citric Acid	5	2.376
Citric Acid	5	2.213	Citric Acid	5	2.297
MeJa	5	1.773	MeJa	5	1.506
MeJa	5	1.622	MeJa	5	1.481
MeJa	5	1.767	MeJa	5	1.372
Chitosan	5	1.919	Chitosan	5	2.408
Chitosan	5	1.987	Chitosan	5	2.284
Chitosan	5	1.847	Chitosan	5	2.668
K_2SO_4	5	1.858	K_2SO_4	5	2.224
K_2SO_4	5	1.953	K_2SO_4	5	2.186
K_2SO_4	5	1.973	K_2SO_4	5	2.246
Control	6	1.854	Control	6	2.364

Control	6	1.901	Control	6	2.298
Control	6	1.835	Control	6	2.325
Citric Acid	6	2.107	Citric Acid	6	2.587
Citric Acid	6	2.022	Citric Acid	6	2.501
Citric Acid	6	1.988	Citric Acid	6	2.482
MeJa	6	1.797	MeJa	6	2.024
MeJa	6	1.800	MeJa	6	2.192
MeJa	6	1.847	MeJa	6	1.871
Chitosan	6	2.238	Chitosan	6	3.425
Chitosan	6	2.355	Chitosan	6	3.245
Chitosan	6	2.203	Chitosan	6	2.944
K_2SO_4	6	2.221	K_2SO_4	6	2.364
K_2SO_4	6	2.398	K_2SO_4	6	2.361
K_2SO_4	6	2.432	K_2SO_4	6	2.321
Control	7	2.099	Control	7	2.507
Control	7	1.978	Control	7	2.413
Control	7	2.086	Control	7	2.402
Citric Acid	7	2.109	Citric Acid	7	2.684
Citric Acid	7	2.552	Citric Acid	7	2.721
Citric Acid	7	2.077	Citric Acid	7	2.697
MeJa	7	1.844	MeJa	7	2.232
MeJa	7	2.016	MeJa	7	2.354
MeJa	7	1.918	MeJa	7	2.198
Chitosan	7	2.304	Chitosan	7	4.067
Chitosan	7	2.115	Chitosan	7	4.082
Chitosan	7	2.232	Chitosan	7	4.061
K_2SO_4	7	2.653	K_2SO_4	7	2.507
K_2SO_4	7	2.632	K_2SO_4	7	2.413
K_2SO_4	7	2.707	K_2SO_4	7	2.321

Wild radish					
20°C			28°C		
Elicitor	Day	Hypocotyl length (cm)	Elicitor	Day	Hypocotyl length (cm)
Control	1	0	Control	1	0
Control	1	0	Control	1	0
Control	1	0	Control	1	0
Citric Acid	1	0	Citric Acid	1	0
Citric Acid	1	0	Citric Acid	1	0
Citric Acid	1	0	Citric Acid	1	0
MeJa	1	0	MeJa	1	0

MeJa	1	0	MeJa	1	0
MeJa	1	0	MeJa	1	0
Chitosan	1	0	Chitosan	1	0
Chitosan	1	0	Chitosan	1	0
Chitosan	1	0	Chitosan	1	0
K_2SO_4	1	0	K_2SO_4	1	0
K_2SO_4	1	0	K_2SO_4	1	0
K_2SO_4	1	0	K_2SO_4	1	0
Control	2	0.592	Control	2	0.522
Control	2	0.489	Control	2	0.402
Control	2	0.532	Control	2	0.586
Citric Acid	2	0.771	Citric Acid	2	0.395
Citric Acid	2	0.848	Citric Acid	2	0.794
Citric Acid	2	0.852	Citric Acid	2	0.817
MeJa	2	0.554	MeJa	2	0.710
MeJa	2	0.690	MeJa	2	0.361
MeJa	2	0.738	MeJa	2	0.519
Chitosan	2	0.748	Chitosan	2	1.235
Chitosan	2	0.551	Chitosan	2	1.136
Chitosan	2	0.633	Chitosan	2	1.156
K_2SO_4	2	0.821	K_2SO_4	2	0.545
K_2SO_4	2	0.745	K_2SO_4	2	0.702
K_2SO_4	2	0.590	K_2SO_4	2	0.586
Control	3	0.883	Control	3	1.034
Control	3	0.850	Control	3	1.101
Control	3	0.905	Control	3	0.943
Citric Acid	3	1.232	Citric Acid	3	1.486
Citric Acid	3	1.236	Citric Acid	3	1.393
Citric Acid	3	1.373	Citric Acid	3	1.290
MeJa	3	0.763	MeJa	3	1.283
MeJa	3	0.888	MeJa	3	1.261
MeJa	3	0.812	MeJa	3	0.971
Chitosan	3	1.003	Chitosan	3	1.741
Chitosan	3	1.153	Chitosan	3	1.836
Chitosan	3	1.033	Chitosan	3	1.905
K_2SO_4	3	1.232	K_2SO_4	3	0.932
K_2SO_4	3	1.105	K_2SO_4	3	0.891
K_2SO_4	3	1.053	K_2SO_4	3	0.876
Control	4	1.245	Control	4	1.306
Control	4	1.395	Control	4	1.387
Control	4	1.230	Control	4	1.421

Citric Acid	4	1.971	Citric Acid	4	1.929
Citric Acid	4	1.658	Citric Acid	4	1.954
Citric Acid	4	1.712	Citric Acid	4	1.886
MeJa	4	1.175	MeJa	4	1.381
MeJa	4	1.279	MeJa	4	1.283
MeJa	4	1.285	MeJa	4	1.454
Chitosan	4	1.362	Chitosan	4	2.432
Chitosan	4	1.465	Chitosan	4	2.003
Chitosan	4	1.315	Chitosan	4	2.321
K_2SO_4	4	1.435	K_2SO_4	4	1.286
K_2SO_4	4	1.593	K_2SO_4	4	1.386
K_2SO_4	4	1.366	K_2SO_4	4	1.575
Control	5	1.446	Control	5	1.844
Control	5	1.378	Control	5	1.734
Control	5	1.507	Control	5	1.898
Citric Acid	5	1.678	Citric Acid	5	3.396
Citric Acid	5	1.688	Citric Acid	5	3.281
Citric Acid	5	1.901	Citric Acid	5	3.100
MeJa	5	1.371	MeJa	5	1.783
MeJa	5	1.602	MeJa	5	1.682
MeJa	5	1.481	MeJa	5	1.752
Chitosan	5	1.336	Chitosan	5	2.704
Chitosan	5	1.502	Chitosan	5	2.321
Chitosan	5	1.515	Chitosan	5	2.486
K_2SO_4	5	1.501	K_2SO_4	5	1.864
K_2SO_4	5	1.522	K_2SO_4	5	1.712
K_2SO_4	5	1.607	K_2SO_4	5	1.738
Control	6	1.577	Control	6	2.837
Control	6	1.465	Control	6	2.687
Control	6	1.597	Control	6	2.754
Citric Acid	6	2.107	Citric Acid	6	3.571
Citric Acid	6	2.022	Citric Acid	6	3.171
Citric Acid	6	1.988	Citric Acid	6	3.482
MeJa	6	1.543	MeJa	6	1.865
MeJa	6	1.555	MeJa	6	1.852
MeJa	6	1.464	MeJa	6	1.901
Chitosan	6	1.786	Chitosan	6	2.976
Chitosan	6	1.803	Chitosan	6	2.604
Chitosan	6	1.899	Chitosan	6	2.792
K_2SO_4	6	1.745	K_2SO_4	6	2.032
K_2SO_4	6	1.688	K_2SO_4	6	2.344

K_2SO_4	6	1.801	K_2SO_4	6	2.176
Control	7	1.840	Control	7	3.137
Control	7	1.853	Control	7	3.212
Control	7	1.760	Control	7	3.253
Citric Acid	7	2.485	Citric Acid	7	3.775
Citric Acid	7	2.538	Citric Acid	7	3.589
Citric Acid	7	2.364	Citric Acid	7	3.597
MeJa	7	1.730	MeJa	7	2.882
MeJa	7	1.762	MeJa	7	2.791
MeJa	7	1.692	MeJa	7	2.940
Chitosan	7	2.452	Chitosan	7	4.450
Chitosan	7	2.573	Chitosan	7	4.490
Chitosan	7	2.607	Chitosan	7	4.360
K_2SO_4	7	2.287	K_2SO_4	7	2.995
K_2SO_4	7	1.750	K_2SO_4	7	3.188
K_2SO_4	7	1.888	K_2SO_4	7	3.086

Table S3. Report on Growth and Bioactive Compound Production in 15-Day-Old *Raphanus sativus* Seedlings at 30°C.

**Treatments**	**Length (cm)**		**Dry Weight (g)**		**Total Glucosinolates (mg 100 g^{-1} DW)**		**Total Anthocyanins (mg 100 g^{-1} DW)**	
	Day 7	*Day 15*	*Day 7*	*Day 15*	*Day 7*	*Day 15*	*Day 7*	*Day 15*
**Control**	2.441cB	3.736bA	4.614cB	7.371bA	53.441bA	30.069bB	3.062bA	1.667bB
**Citric Acid**	2.701bB	3.663bA	8.088aB	11.577aA	57.996bA	27.377bB	2.231bA	1.318bB
**MeJa**	2.261dB	4.159bA	5.068bcB	8.250bA	70.791abA	46.051aB	5.362aA	4.073aB
**Chitosan**	4.070aB	5.663aA	7.027abB	10.880aA	66.824abA	47.817aA	2.124bA	0,835bB
**K_2SO_4**	2.414cdB	3.517bA	3.521cB	7.565bA	83.506a A	58.488aB	2.192bA	1.643bB

Different lower case letters indicate statistically significant differences between treatments. Different capital letters indicate statistically significant differences between days. Mean separation within a column followed by different letters are significantly different according to Tukey test at $p \leq 0.05$.

Table S4. Report on Growth and Bioactive Compound Production in 15-Day-Old Raphanus raphanistrum Seedlings at 30°C.

**Treatments**	**Length (cm)**		**Dry Weight (g)**		**Total Glucosinolates (mg 100 g^{-1} DW)**		**Total Anthocyanins (mg 100 g^{-1} DW)**	
	Day 7	*Day 15*	*Day 7*	*Day 15*	*Day 7*	*Day 15*	*Day 7*	*Day 15*
**Control**	3.201cB	4.113bA	1.812cB	4.506cA	44.818bA	25.546bB	3.063bA	2.144bA
**Citric Acid**	3.654bB	4.458bA	3.357aB	7.210aA	53.570bA	30.535bB	2.424bA	1.697bB
**MeJa**	2.871dB	3.903bA	2.100bcB	5.454bcA	73.881aA	42.112aB	5.362aA	3.753aB
**Chitosan**	4.433aB	5.200aA	2.872abB	6.243bA	73.162aA	41.702aB	2.124bA	1.487bA
**K_2SO_4**	3.090cdB	3.990bA	2.203bcB	5.773bA	91.478aA	52.142aB	2.192bA	1.535bB

Different lower case letters indicate statistically significant differences between treatments. Different capital letters indicate statistically significant differences between days. Mean separation within a column followed by different letters are significantly different according to Tukey test at $p \leq 0.05$.

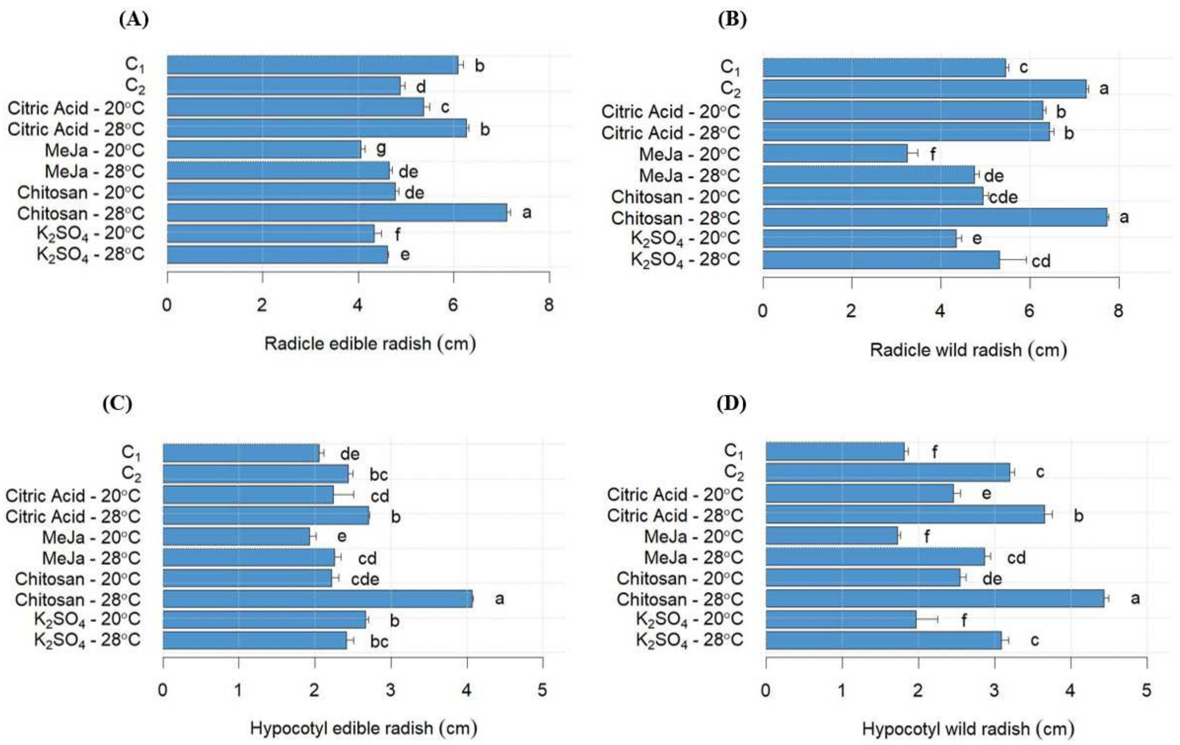

Figure S1. Effect of elicitors on radicle growth and hypocotyl growth in 7-day germinated sprouts. Letters A and B represent the Hypoctyl growth of ER and WR, respectively. Letters C and D represent the radicle growth of ER and WR, respectively. C1: Control at 20°C; C2: Control at 30°C; Citric Acid – 20°C represents the citric acid elicitor treatment combined with temperature at 20°C; Citric Acid – 30°C represents the citric acid elici-tor treatment combined with temperature at 30°C; MeJa – 20°C represents the MeJa elicitor treat-ment combined with temperature at 20°C; MeJa – 30°C represents the MeJa elicitor treatment combined with temperature at 30°C; Chitosan – 20°C represents the chitosan elicitor treatment combined with temperature at 20°C; Chitosan – 30°C represents the chitosan elicitor treatment combined with temperature at 30°C; K2SO4 – 20°C represents the sulphate potassium elicitor treatment combined with temperature at 20°C; K2SO4 – 30°C represents the sulphate potassium elicitor treatment combined with temperature at 30°C. Different letters mean significant differ-ences at $p < 0.05$ in treatments for radish sprouts analyzed separately (edible and wild radish) according to Tukey test.

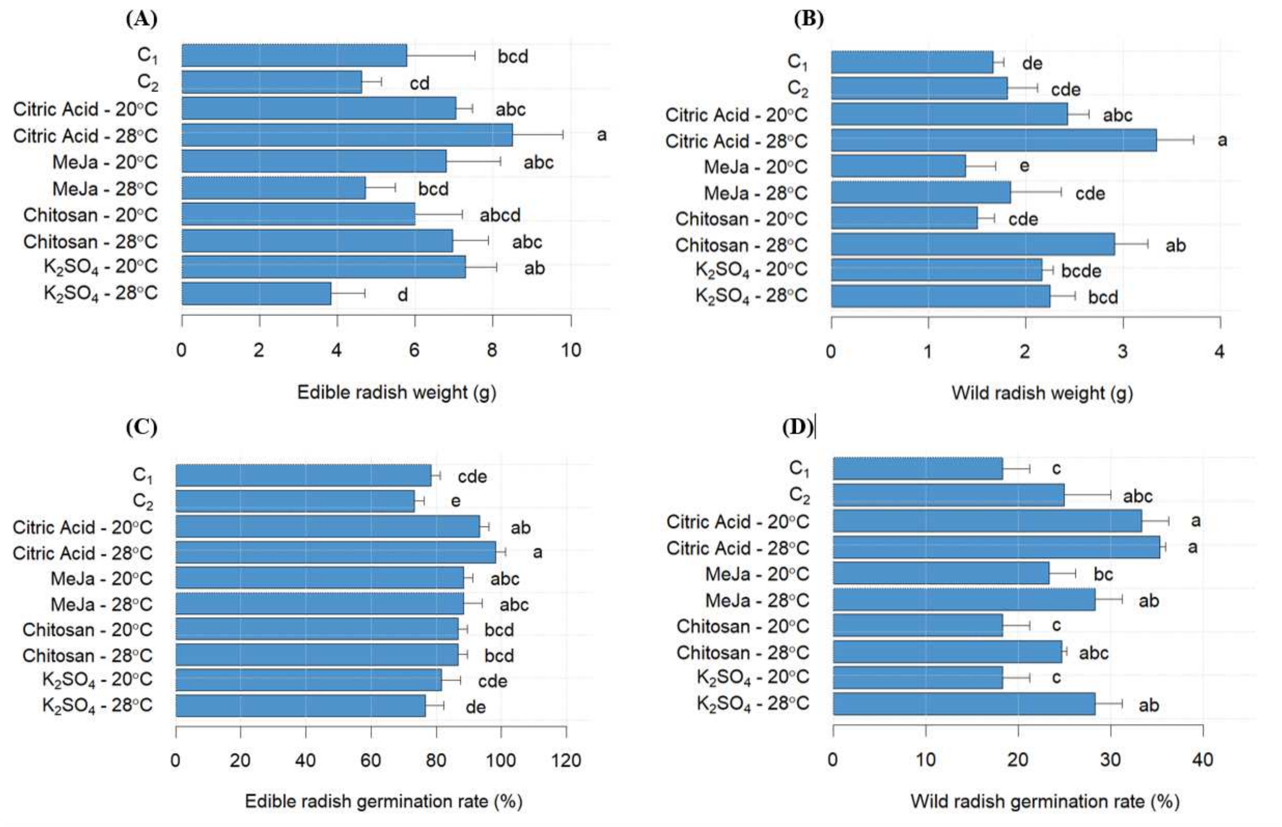

Figure S2. Effect of elicitors on fresh weight and germination rate in 7-day germinated sprouts. Letters A and B represent the fresh weight of ER and WR. Letters C and D represent the germination rate of ER and WR. C1: Control at 20°C; C2: Control at 30°C; Citric Acid – 20°C represents the citric acid elicitor treatment combined with temperature at 20°C; Citric Acid – 30°C represents the citric acid elici-tor treatment combined with temperature at 30°C; MeJa – 20°C represents the MeJa elicitor treat-ment combined with temperature at 20°C; MeJa – 30°C represents the MeJa elicitor treatment combined with temperature at 30°C; Chitosan – 20°C represents the chitosan elicitor treatment combined with temperature at 20°C; Chitosan – 30°C represents the chitosan elicitor treatment combined with temperature at 30°C; K2SO4 – 20°C represents the sulphate potassium elicitor treatment combined with temperature at 20°C; K2SO4 – 30°C represents the sulphate potassium elicitor treatment combined with temperature at 30°C. Different letters mean significant differ-ences at $p < 0.05$ in treatments for radish sprouts analyzed separately (edible and wild radish) according to Tukey test.

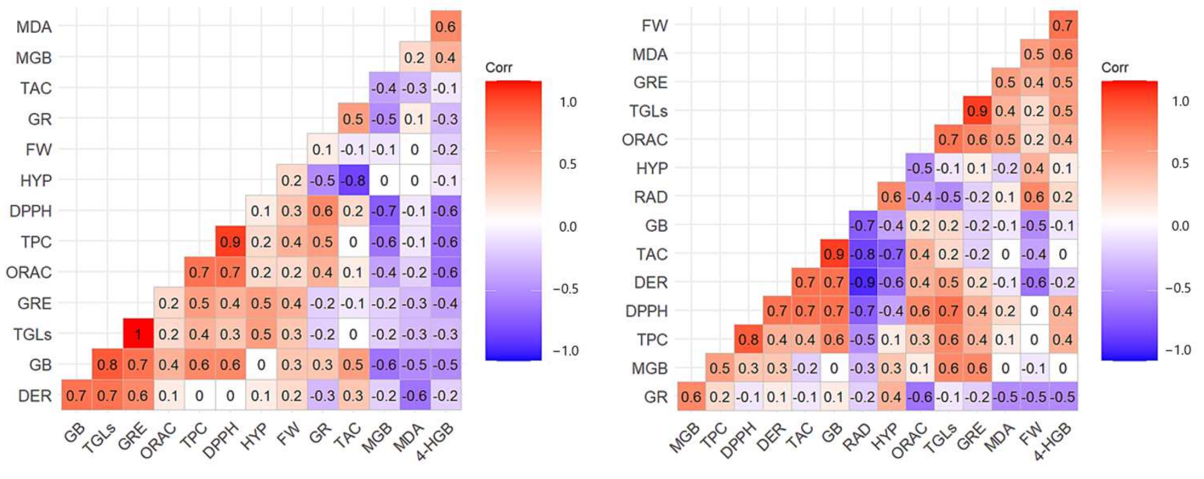

Figure S3. Correlation matrix between different variables at 20°C, (A) correspond to ER and (B) correspond to WR.

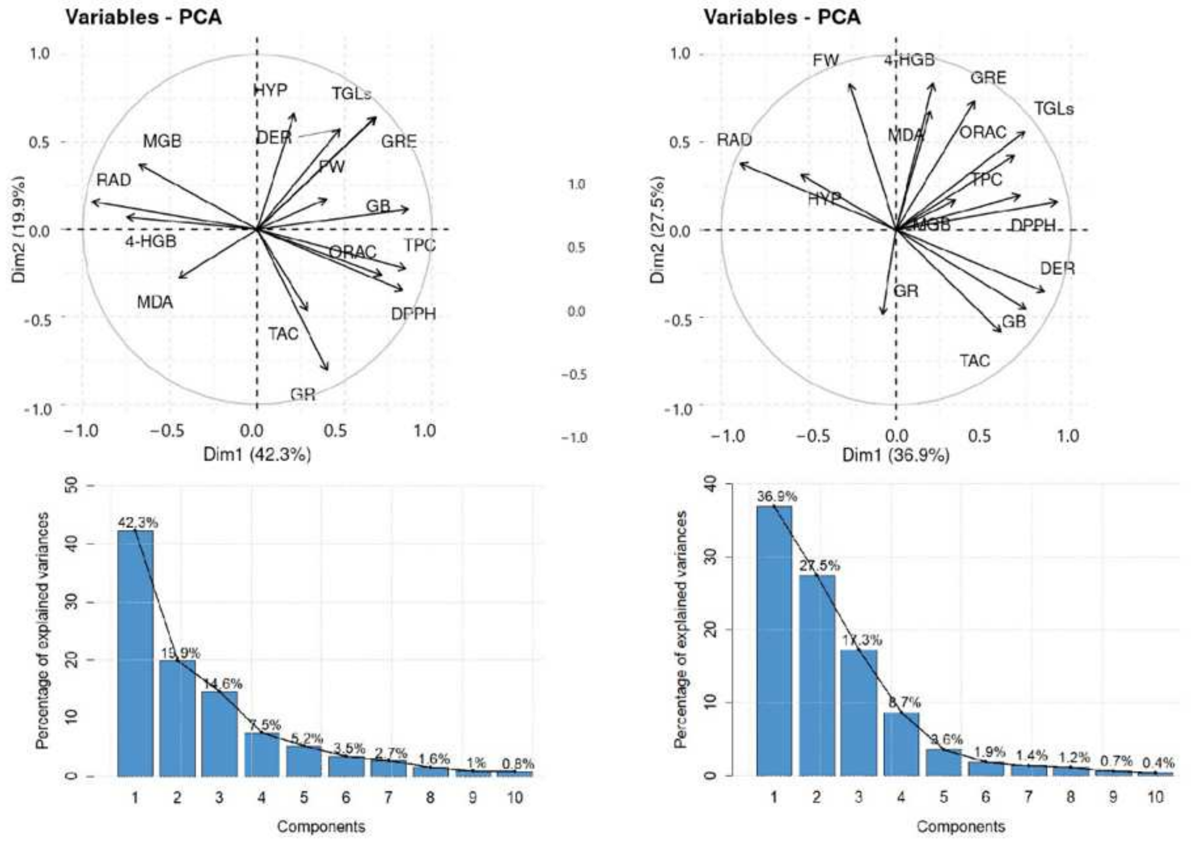

Figure S4. Principal component analysis (PCA) at 20°C. Letter (A) represent the PCA of edible radish, letter (B) represent the PCA of wild radish, letter (C) represent the percentage of explained of edible radish and letter (D) represent the percentage of explained of wild radish. RAD: Length of the radicle; HYP: Hypocotyl length, FW: weight sprouts; GR: germination rate; MDA: Malondialdehyde Assay; TGLs: Total Glucosinolates; TAC: Total anthocyanins; GRE: Glucoraphenin; 4-HGB: Hydroxyglucobrassicin; DER: Dehydroerucine; GB: Glucobrassicin; MGB: 4-methoxyglucobrassicin; TPC: Total phenolic content; DPPH: DPPH assay; ORAC: ORAC assay for ER and WR.

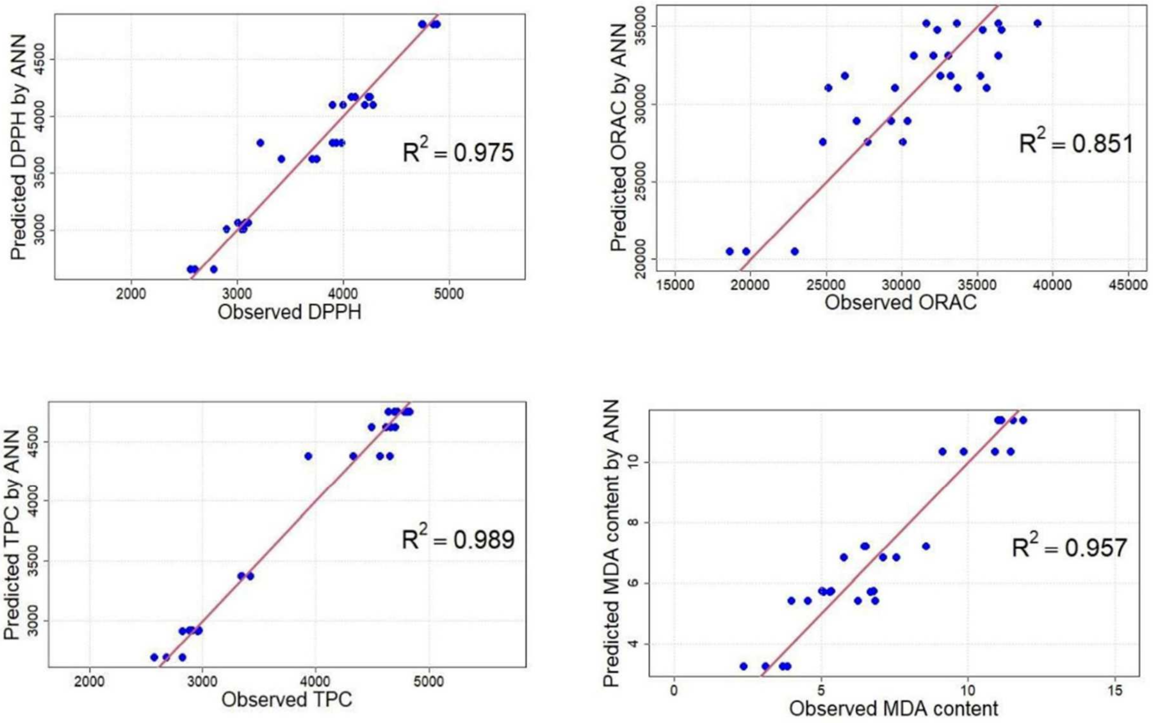

Figure S5. Variables predicted by ANNs v/s observed variables for DPPH, ORAC, TPC and MDA.
